# Unraveling the Molecular Composition and Reactivity Differentiation of Algae- and Macrophyte-Derived Dissolved Organic Matter in Plateau Lakes: Insights from Optical Properties and High Resolution Mass Spectrometry Characterization

**DOI:** 10.3390/molecules30173510

**Published:** 2025-08-27

**Authors:** Qiuxing Li, Runyu Zhang, Haijun Yuan, Liying Wang, Shuxia Xu

**Affiliations:** 1 College of Earth and Planetary Sciences, Chengdu University of Technology, Chengdu 610059, China; liqiuxing1021@163.com (Q.L.); xushux@cdut.edu.cn (S.X.); 2 State Key Laboratory of Environmental Geochemistry, Institute of Geochemistry, Chinese Academy of Sciences, Guiyang 550081, China; yuanhaijun@mail.gyig.ac.cn (H.Y.); wangliying@vip.gyig.ac.cn (L.W.); 3 University of Chinese Academy of Sciences, Beijing 100049, China; 4 College of Ecology and Environment, Chengdu University of Technology, Chengdu 610059, China

**Keywords:** dissolved organic matter, FT-ICR MS, Orbitrap MS, low molecular weight, molecular composition, eutrophication

## Abstract

Most lacustrine dissolved organic matter (DOM) still lacks comprehensive environmental sources and molecular characterization, especially in plateau lakes. Herein, macrophytes and algae from contrasting lakes of the Yunnan-Guizhou Plateau, together with Suwannee River fulvic acid (SRFA), were used to characterize the total identified DOM (Bulk-DOM) and low-molecular-weight DOM (LMW-DOM, <200 Da). To address this, we combined spectroscopy with Fourier transform ion cyclotron resonance (FT-ICR) and Orbitrap mass spectrometry (MS). Algae-derived DOM (ADOM) exhibited endogenous DOM characteristics, while macrophyte-derived DOM (MDOM) showed the characteristics of endogenous and terrigenous DOM. ADOM contained numerous heteroatoms, with high proportions of proteins, carbohydrates, and lipids. The chemical structures of ADOM were more aliphatic and degradable than that of MDOM. Conversely, MDOM and SRFA had higher degree of humification and aromaticity and showed greater resistance to microbial degradation. The capability of Orbitrap MS to characterize P-containing molecules was superior to FT-ICR MS. Moreover, significant differences were found between FT-ICR and Orbitrap MS in weighted average carbon atom number, weighted average mass-to-charge ratio, carbohydrates, and P-containing compounds. LMW-DOM accounted for approximately 10% of Bulk-DOM. Compared to Bulk-DOM, LMW-DOM was more active than Bulk-DOM because of the reduced state and more N-containing compounds. This study provides a valuable perspective to reveal the molecular characteristics and behaviors of ADOM and MDOM, which has crucial implications for carbon cycling in aquatic ecosystems.

## 1. Introduction

Dissolved organic matter (DOM) serves as an active carbon pool on the Earth’s surface [1] and profoundly impacts the structure and function of aquatic ecosystems [2]. Especially in plateau lakes, DOM has emerged as a key controlling factor in ameliorating eutrophication and hindering algal blooms [3]. DOM not only provides nutrients and energy for organisms but also transports elements such as carbon (C), nitrogen (N), and phosphorus (P) into aquatic environment. Accordingly, the spectral characteristics of DOM are conducive to identifying the lake eutrophication [2]. Plateau lakes have long been recognized as ideal settings for DOM transformation, showing sensitivity to variations of climates (e.g., light and temperature) and microbes [4]. There is evidence that DOM derived from plateau lakes inherently shows a tight linkage with the carbon biogeochemical cycles worldwide [5]. More specifically, the processing of DOM can mineralize organic carbon and generate carbon dioxide, thereby exacerbating the global carbon cycle [3]. In China, the number and area of plateau lakes account for 67% and 74% of total lakes, respectively [6]. However, there is currently limited knowledge about the molecular properties of DOM and its biogeochemical reactivity in plateau lakes.

Algae and macrophytes are hotspots of aquatic DOM, whose transformation and regeneration largely threaten or impede ecological health [1]. As the primary contributors of endogenous DOM, the release of algae-derived DOM (ADOM) and macrophyte-derived DOM (MDOM) is inextricably linked with lake eutrophication [7]. For instance, ADOM fundamentally comprises N- and S-rich lipids, peptides, and polysaccharide derivatives, while MDOM is dominated by aromatic compounds such as lignin, phenols, and tannins [8]. Recent studies have demonstrated that low-molecular-weight DOM (LMW-DOM) and heteroatom (e.g., N, S, P) in DOM exhibit high bioavailability and reactivity in aquatic ecosystems [9]. As such, LMW-DOM (e.g., amino acids) often serves as the preferred substrate for microbial metabolism due to its high bioactivity [2]. More interestingly, S-containing DOM is pivotal to heavy metal complexation and photochemical reactions [10], while P-containing DOM has a crucial role in regulating the potential mechanisms of microbially mediated P cycling and lake eutrophication [11]. Nevertheless, the presence of DOM in aquatic environments is often highly underestimated due to its chemical instability (e.g., photodegradation or biodegradation) and ionization inhibition effects [5]. Considering the dramatic threat of eutrophication to plateau lakes, it is crucial to decipher the molecular properties of algae and macrophyte DOM and their lability or persistence.

The composition and sources of DOM can generally be characterized by isotopic tracing, optical spectroscopy, and high-resolution mass spectrometry [12]. Advanced fluorescence spectroscopy techniques like excitation–emission matrix spectrum (EEMs) with parallel factor analysis (PARAFAC) can rapidly assess the origin source and transformation process of DOM by delineating the fluorescent components (e.g., humic acid-like, protein-like components) [13]. The application of Fourier transform ion cyclotron resonance mass spectrometry (FT-ICR MS) has achieved significant advancements in the field of molecular composition analysis of DOM [14]. Ultra-high resolution (>500,000) and mass accuracy are the basis for accurate calculation of the DOM formula [15]. Prior study has revealed the spatial variation in molecular composition of DOM in 22 plateau lakes across China by implementing FT-ICR MS [16]. Unlike FT-ICR MS, Orbitrap mass spectrometry (Orbitrap MS) exhibits distinct technological advantages in dynamically tracking DOM molecular changes by preserving high resolution (>240,000 at *m*/*z* 200). Meanwhile, integrating rapid scanning capability and high sensitivity makes Orbitrap MS particularly suitable for high-throughput analysis of complex DOM samples [17]. Since the Orbitrap MS can extend the mass to 50 Da, it is more preferable for characterizing the segment composition of LMW-DOM than FT-ICR MS [9]. Based on these high-resolution MS technologies, it is necessary to better understand the composition and origin of active DOM, such as LMW-DOM and heteroatom DOM.

Here, we collected algae and macrophytes from three contrasting lakes on the Yunnan-Guizhou Plateau, Southwestern China. These samples were optically characterized by combining ultraviolet-visible absorption spectroscopy (UV-Vis) and EEMs and qualitatively analyzed by FT-ICR MS and Orbitrap MS. This study aims to (1) systematically reveal the composition characteristics of DOM molecules (e.g., molecular formula, heteroatom distribution, small molecule distribution, etc.) in plateau lakes; (2) explore the association mechanisms of algal and macrophyte DOM with their sources (e.g., endogenous or terrigenous) and reactivity differentiation; and (3) investigate the differences between FT-ICR MS and Orbitrap MS in characterization of aquatic DOM. It is challenging to analyze the differences of DOM from different sources at the molecular level, so we are trying to obtain the possible molecular structure and formation mechanism of DOM. This study overcomes the limitations of conventional single characterization techniques and provides a comprehensive molecular-level perspective for the source resolution and ecological effect assessment of lake DOM.

## 2. Results and Discussion

### 2.1. Elemental Distribution of Macrophytes and Algae

Macrophytes and algae had similar total organic carbon (TOC) content, ranging from 36 to 50%, while Suwannee River fulvic acid (SRFA, 2S101F) exhibited the highest value of 52% (Table S1). These differences are highly consistent with previous findings [8,18]. Conversely, the total nitrogen (TN) content of algae (4.46–7.30%) was significantly higher than that of macrophytes (0.96–4.38%) and SRFA (0.67%). Prior studies have reported that the TN content of DOM in soils ranged between 0.5 and 3.7% [19], which is remarkably lower than our results (particularly in ADOM). This is reasonable because numerous free-living algae like Cyanobacteria exhibit a unique capability to fix nitrogen from the air, thus achieving an increase in TN [20].

The C/N value is a valuable indicator to discriminate the source of DOM [20]. SRFA exhibited the highest C/N molar ratio (91.1), followed by macrophytes (10–48) and algae (6–10) (Table S1). This observation is in coincidence with earlier reports that the C/N ratio of terrestrial plants is generally higher than 30 [21]. In contrast, the C/N ratio of lower plants is typically less than 10, with average values for zooplankton (6.3), phytoplankton (6.0), diatoms (5.5 to 7.0), blue-green algae (6.5), and benthos (2.8 to 3.4) [22]. Our study indicated that algae had distinctive endogenous DOM characteristics. The C/N ratio of macrophytes is probably a mixture of both the endogenous and exogenous sources [8]. As for the C/N ratios, macrophytes were intermediate between algae and terrestrial DOM, which arose from the absence of cellulose in algae and its abundance in vascular plants [13].

### 2.2. Spectral Properties of Macrophytes and Algae

#### 2.2.1. UV-Vis Absorption Spectral Parameters Analysis

The absorbance at 254 nm (a254) and at 300 nm (a300) usually indicate the concentration of chromophoric dissolved organic matter (CDOM) and the amount of aromatic compounds in aquatic DOM [13]. Specifically, the a254 is often employed to quantify the extent of unsaturation in DOM [23]. For a254 and a300, SRFA exhibited the highest values, followed by macrophytes (e.g., water hyacinth (WH), myriophyllum verticillatum (MV), and hydrilla verticillata (HV)) and algae (Erhai algae (EA), Puding algae (PA), and Dianchi algae (DA)) (Table S2). Our findings suggested that the terrestrial DOM represented by SRFA exhibited a higher CDOM level, while the endogenous DOM represented by algae had a lower CDOM level. This is supported by a previous study showing that terrestrial plants exceed macrophytes and submerged plants exceed algae with regard to CDOM concentrations [24]. The degree of humification is often indicated by absorbance ratios at 300 to 400 nm (E3/E4), 250 to 365 nm (E2/E3), and 465 to 665 nm (E4/E6), where E4/E6 can also reflect the aromaticity of DOM [25]. SRFA, WH, and MV exhibited elevated levels of E3/E4 (>4), E2/E3 (>4) and E4/E6 (>2.5). According to the published literature [21], the order of magnitude of humification for macrophytes was generally as follows: floating plants > submerged plants > aquatic plants. Moreover, submerged plants are typically higher than planktonic algae [21].

The slope ratio (SR) value reflects the DOM properties, including molecular weight, endogenous or terrigenous characteristics, and photochemical reactivity [13,21]. For instance, a low SR value indicates high molecular weight, high aromaticity, high photochemical reactivity, and vascular plant-like organic matter [26]. The SR values of SRFA, WH, and MV were all less than 1 (Table S2), indicating exogenous sources. Conversely, SR values of ADOM and HV were greater than 1, predominantly attributed to endogenous autobiological action in aquatic ecosystems. As reported in a previous study [27], algae SR is 1.79 ± 0.52, while macrophyte SR is 0.35 ± 0.58. The difference can be explained that algae do not contain cellulose, while macrophytes are rich in cellulose. As such, the SR of macrophytes is much lower than that of algae. Furthermore, the SR values indicated the higher molecular weight and photodegradation activity of MDOM than those of ADOM. It has been widely reported that lignin/CRAM-like structures and aromatic structures in MDOM are readily photodegradable [15].

#### 2.2.2. Three-Dimensional EEMs Fluorescence Spectrum Analysis

**Figure 1:**
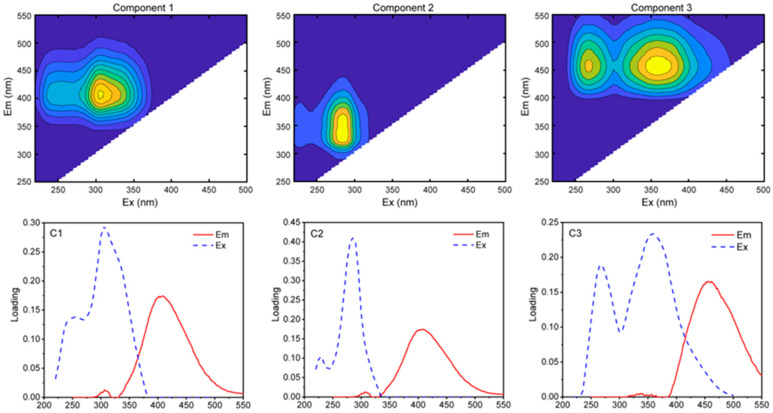
Three fluorescence components identified by the 3D-EEM-PARAFAC model in different DOM samples.

Three major fluorescence components were identified by PARAFAC modeling and further validated through split-half analysis (Figure 1). Thereinto, C1 (excitation wavelength (Ex)/excitation wavelength (Ex) of 315 nm/422 nm and 250 nm/422 nm) is a fulvic acid fluorescence component derived from terrestrial source, C2 (Ex/Em of 280 nm/354 nm) is a typical protein-like fluorescence component and belongs to endogenous organic matter, while C3 (Ex/Em 260 nm/446 nm and 345 nm/446 nm) is a humic acid-like component [28,29]. As shown in Table S3, C1 accounted for the largest proportion in SRFA (52%), followed by macrophytes (14–36%). C2 held the highest abundance in algae (75–81%), followed by macrophytes (45–82%) and SRFA (only 4%). C3 was the most abundant in SRFA, followed by algae and macrophytes.

Algae exhibited the highest proportion for C2, reflecting the great potential contribution of endogenous DOM, which is in line with the literature [21,23]. This result is also confirmed by a_254_ and a_300_ presented in Table S2 above. It is well known that humic acid-like and fulvic acid-like fluorescence components (e.g., C1 and C3 components) primarily originate from terrestrial plant metabolism or microbial metabolism, whereas protein-like or amino acid-like substances mainly originate from endogenous organic matter sources in aquatic environments [8,13,30].

Fluorescence indices such as fluorescence index (FI), humification index (HIX), and biological index (BIX) offer a valuable insight into the chemical composition and biological activities of different sources of DOM. Among them, the FI can reveal the source of DOM: when FI > 1.9, the sources are mainly from microbial activities and dominated by endogenous inputs; when FI < 1.4, the sources are predominantly from terrigenous DOM [31]. In this study, the lowest FI value (1.1) reflected the unique characteristics of terrigenous DOM in SRFA, while a higher FI value (>2.1) indicated the typical characteristics of endogenous DOM in algal (Table S3). In contrast, the FI value of macrophytes ranged from 1.5 to 1.7, substantially exhibiting the mixed characteristics of endogenous and terrestrial sources [8]. The HIX index can identify the degree of humification or maturity of organic matter. For DOM, the higher the degree of its humification, the better its stability and the longer its existence in the environment [9]. Generally, SRFA exhibited high HIX values (>10), while macrophytes and algae were characterized by low HIX values (<2) (Table S3). The BIX can reflect the high or low bioavailability of endogenous DOM. Elevated BIX value indicates the presence of fresh DOM input and its high bioavailability [32]. When BIX > 1, the nascent endogenous DOM’s primary contribution was to freshwater lakes. When BIX is between 0.6 and 0.7, the proportion occupied by endogenous DOM is relatively small. For BIX, except for HV, which was greater than 1, algae and WH were slightly less than 1 (Table S3). Such deviation may be attributed to the fact that the samples have undergone varying degrees of biodegradation during sampling, transportation, and pretreatment. For instance, algae are usually kept in cold storage for 5 days after sampling and then transferred to the laboratory for isolation and characterization. In contrast, owing to its mature root system and larger biomass, MV required a longer freeze-drying time than other macrophytes. A prior study reported that the BIX of macrophytes slightly decreased within 5 days and then further decreased, whereas the BIX of algae decreased sharply within 5 days and fluctuated at different time intervals [27].

### 2.3. FT-ICR MS and Orbitrap MS Analysis of Bulk-DOM

#### 2.3.1. Mass Spectral Parameters Analysis of Bulk-DOM

The mass spectra of FT-ICR MS and Orbitrap MS in the negative ion mode are shown in Figure S1. As for FT-ICR MS, the majority of cumulative ion abundance was concentrated in a much narrower mass range (*m*/*z* 200–600) in all samples. The broadband mass spectrum produced by Orbitrap is quite different from that of FT-ICR-MS, mainly clustered in the range of 100–400 *m*/*z*. This is expected because of the ubiquitous discrimination of low *m*/*z* ions in FT-ICR MS [33,34,35]. In contrast, the advantage of Orbitrap MS on low-mass molecule characterization has become increasingly evident in numerous studies [9,17,34,36]. These LMW-DOM components are easier to ionize and detect than HMW-DOM in Orbitrap MS [37].

Furthermore, the parameters following molecular formula assignment by the FT-ICR MS analysis of differing samples are summarized in Table 1. The normalized average molecular weights (*m*/*z*_wa_) ranged from 350 to 440 Da. The number of molecular formulae assigned to the samples ranged from 1514 to 7817, with a greater number of ADOM than MDOM. The lowest molecule number identified in MV may be ascribed to the presence of numerous metal elements, such as Ca and Fe in the extracted DOM, influencing its response to the MS analysis [38]. The percentage of C_wa_ was consistent, even (17–21%) among DOM samples, whereas O_wa_ exhibited higher levels in WH and MV. Conversely, algae and HV showed higher levels of H_wa_, N_wa_, and S_wa_. The proportions of P_wa_ and P cpd. were also higher in ADOM from EA and PA, which aligns with the results of the elemental composition. The molecular composition of DOM in all samples was dominated by CHO, and the proportion was significantly higher in MDOM than in ADOM. Whatever the elemental composition and molecular properties, HV was very similar to algae because of certain commonalities in the physiological characteristics of both growing in aquatic environments [39].

Compared with the prior study in eutrophic shallow Taihu Lake (H/C_wa_ = 1.29, O/C_wa_ = 0.55) [8], the H/C_wa_ of MV in plateau lakes (1.02) was slightly lower, while the O/C_wa_ (0.56) was highly consistent. The H/C_wa_ and O/C_wa_ of algae fell within the ranges of 1.30–1.67 and 0.23–0.40, respectively, both of which have been reported previously in the literature [7,8,40,41,42]. Referring to previous studies [30], low O/C_wa_ and high H/C_wa_ of algae and HV revealed the remarkable characteristics of low molecular weight, low oxygen content, and high saturation of endogenous DOM. In contrast, WH, MV, and SRFA exhibited high O/C_wa_ and low H/C_wa_, reflecting the nature of high molecular weight, high oxygen content, and low saturation of terrestrial DOM (Figure S2a). WH, MV, and SRFA showed greater DBE_wa_ and AI-mod_wa_, suggesting that their DOM were more unsaturated and aromatic. Meanwhile, WH and MV possessed larger I_DEG_ and CRAM, and smaller MLBL, indicating that these DOM were relatively older, more stable, and less susceptible to degradation. This finding was verified by the HIX index in EEMs. Furthermore, their positive NOSC values indicated that the molecules were in an oxidized state. In comparison, DOM from algae and HV have smaller DBE_wa_ and AI-mod_wa_, indicating their lower unsaturation and aromaticity, which supported the results of a_254_ and S_R_ in UV-Vis analysis. Besides, the samples exhibited lower I_DEG_, lower CRAM (29–35%) and larger MLBL (54–61%), thereby confirming their younger age, molecular instability, and susceptibility to microbial degradation and photochemical reaction. The negative NOSC indicated that the molecules were in the reduced state and susceptible to degradation [43]. The literature has also documented that S-containing substances reduce carbon chain length and saturation, and are prone to reduction, thereby decreasing the stability of organic matter [43]. Consequently, ADOM exhibited a higher degradation rate and efficiency than MDOM [44].

Besides this, the Orbitrap MS were roughly similar to those of the FT-ICR MS (Table S4). The 2405–4010 molecular formulas assigned by Orbitrap MS were less than those by FT-ICR MS, except for MV. Some of these were not determined primarily because of the resolution power and other reasons like ionization efficiency or detection limit [33,34]. In addition, some ion peaks were either lost, probably in Orbitrap MS, due to insufficient resolution of small peaks or overlaped by larger and neighboring signals at mass ranges above *m*/*z* 300 in comparison with FT-ICR MS [36].

Compared to FT-ICR MS, Orbitrap MS had lower *m*/*z*_wa_, C_wa_, H_wa_, O_wa_, DBE_wa_, I_DEG_, and CHO cpd. (%). There were remarkable differences between FT-ICR MS and Orbitrap MS in molecule numbers and the relative proportions of molecules containing N/S/P heteroatoms. FT-ICR MS responded more to S, while Orbitrap MS responded more to P. Notably, both FT-ICR MS and Orbitrap MS responded more or less equally to N. According to Orbitrap MS analysis, the H/C of ADOM and HV remained relatively stable, exhibiting a shift toward high O/C (Figure S2b). In contrast, WH, MV, and SRFA moved toward high H/C and low O/C, exhibiting a greater magnitude of variation. This is presumably attributed to the inability of organic compounds with high oxygen content in Orbitrap MS to be ionized, inhibiting the ionization processes [45]. The negative NOSC values suggested that Orbitrap MS may be more conductive to detecting compounds in the reduced state [46].

A comparison of DOM composition by FT-ICR MS and Orbitrap MS has demonstrated that *m*/*z*_wa_ and O/C_wa_ for SRFA are slightly different between the two high resolution MS [34]. It is important because all even *m*/*z* assignments (in negative mode) mainly depend on resolution [33]. Given that Orbitrap MS is largely limited by low-resolution MS instruments, certain arrangements of heteroatoms, including most N, S, and P-containing formulae, should not be considered [47]. Currently, these homologous series can be easily resolved at resolution mode above 100,000 for Orbitrap MS, particularly for the discrimination of DOM with higher heteroatom content (i.e., more number of formulas containing N, S, or P) [36]. Numerous studies have demonstrated that drawbacks in resolving power can be addressed by increasing the specificity for analytes during extraction [48], ionization [45], or instrumental detection [49]. Collectively, the molecular structure and compositional characteristics of DOM from ADOM and MDOM exhibited significant heterogeneity, irrespective of the high-resolution MS technique employed.

#### 2.3.2. Molecular Composition of Bulk-DOM

**Figure 2:**
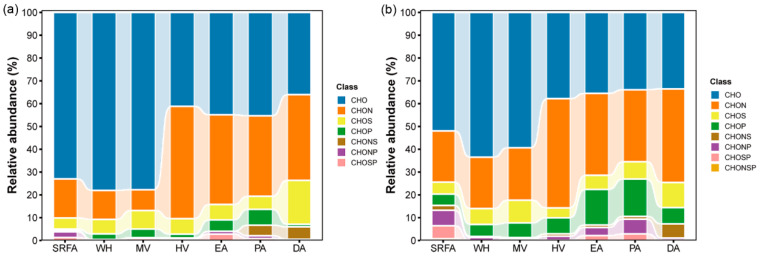
Composition of major subcategories of DOM in various samples tested by (**a**) FT-ICR MS and (**b**) Orbitrap MS.

We further compared the difference in molecular composition of DOM between MDOM and ADOM. The ADOM showed similar proportions for CHO (36–45%) and CHON (35–40%) fractions, and it also contained CHOS, CHONS, and CHOP for the FT-ICR MS analysis. Note that CHO fractions (73–78%) were predominant in WH, MV, and SRFA, followed by CHON (9–13%), CHOS (6–8%), and CHOP (2–4%) (Figure 2a). This contrasts sharply with a recent study on Taihu Lake using FT-ICR MS analysis [8]. It is worth mentioning that a higher proportion of S-containing compounds (9%) was identified, and previously undetected P-containing compounds were detected in ADOM in the present study (Table S5). Compared with the literature, a higher *m*/*z*_wa_ was obtained in the DOM from MV.

Intriguingly, ADOM showed higher total proportions (approximately 55–64%) of N-, S-, and P-containing molecules originating from in-lake metabolic pathways. The N fraction in algae was primarily composed of CHON, CHONS, and CHONP. Previous studies have reported that the proportion of N, S, and P molecules in the DOM of the Yangtze River reaches 56.6% [50]. Large amounts of N-containing formulas (i.e., CHON and CHONS molecules) are contained in the ADOM, accounting for 51.8% of the total formulas [51].

The main form of S-containing compounds was identified as C_c_H_h_O_3–7_S, and the molecule numbers of CHOS and CHONS in ADOM were greater than those in MDOM and SRFA. Particularly, FT-ICR MS and Orbitrap MS consistently showed that the number of O_3_S + O_5_S compounds was the highest in DA, followed by PA and EA (Table S6). Previous studies have pointed out that the S-containing compounds in DOM increase with the increase in lake eutrophication [12]. A recent report found that the decomposition of cyanobacteria can release large amounts of sulfur compounds [52]. The increase in the S-containing components in DOM corresponds to a rise in its instability in the reduced state by reducing the length of the carbon chain and the saturation degree of DOM. Therefore, the higher relative abundance of O_3_S and O_5_S in ADOM in this study may exacerbate the mineralization degradation of DOM [43].

P-containing compounds exhibited a high proportion of CHOP in MDOM and ADOM (Tables S7 and S8). These components had relatively simple structures and poor stability. In contrast, the P-containing components of SRFA were dominated by CHONP, followed by CHOSP. These components had more complex molecular structures, a high degree of molecular condensation, and were usually difficult to degrade. The prevalence of DOP in surface water has been documented in the extant literature, where the percentage of identified P-containing molecular formulas is less than 1% [53]. Conversely, our MS parameters and molecular formula matching process were more suitable for the identification of P-containing compounds.

Subsequent examination of labile compounds (i.e., MLBL (%)) in ADOM and MDOM indicated that CHOS compounds had the highest MLBL (%) of 72–97%, followed by CHOP compounds (40–81%), CHO compounds (14–58%), and CHON compounds with the lowest MLBL (%). This finding further confirmed a higher lability of S- and P-containing compounds compared to N-containing compounds [14]. The higher MLBL (%) indicated that S- and P-containing compounds in DOM were more chemically reactive and bioavailable. What is more, Orbitrap MS was superior to FT-ICR MS in terms of its capacity to characterize S- and P-containing molecules and to resolve a greater number of compound types, specifically CHONSP (Figure 2b). This may be related to the distinct composition of DOM samples, instrumental performance, matching strategies, etc. [36,54].

#### 2.3.3. Van Krevelen Plots of Bulk-DOM in Macrophytes and Algae

**Figure 3:**
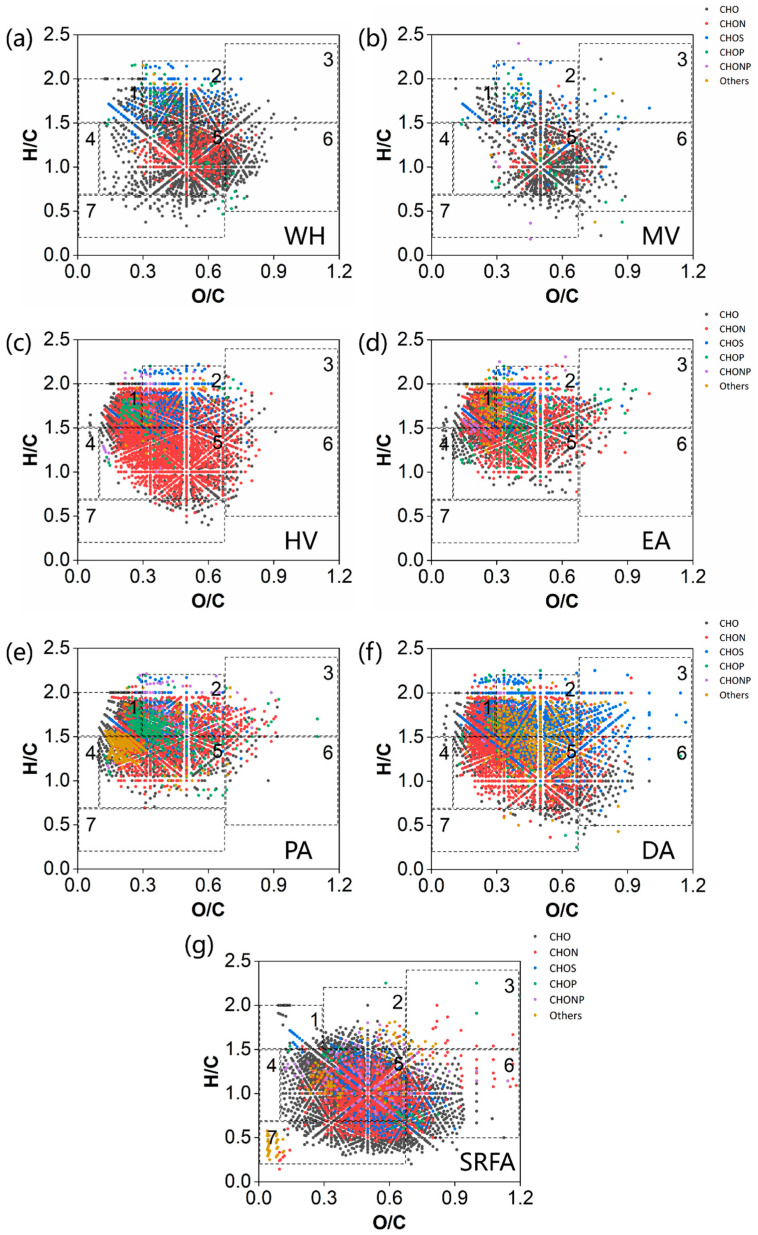
VK maps of compounds in macrophytes, algae, and SRFA via FT-ICR MS. (**a**) WH, (**b**) MV, (**c**) HV, (**d**) EA, (**e**) PA, (**f**) DA, and (**g**) SRFA. The numbers in the figure represent (**1**) lipids, (**2**) proteins (including peptides), (**3**) carbohydrates, (**4**) unsaturated hydrocarbons, (**5**) lignins, (**6**) tannins, and (**7**) condensed aromatic molecules.

**Figure 4:**
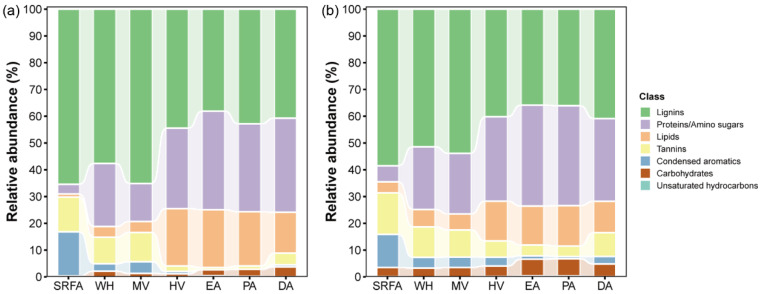
Relative abundance (%) of van Krevelen diagram-derived classification of DOM from various sources by (**a**) FT-ICR MS and (**b**) Orbitrap MS.

The Van Krevelen (VK) plots of SRFA, characterized by FT-ICR MS and Orbitrap MS, are shown in Figure S3. SRFA and MDOM showed high concentrations of lignins, tannins, and condensed aromatic molecules (Figure 3). The large percentage of lignin/CRAM and tannin compounds observed for SRFA (Figure 4) was in line with earlier high-resolution MS measurements [17,40]. In contrast, ADOM exhibited higher abundance of proteins, lipids, and carbohydrates (Table S9). The above results were consistent with AI-mod_wa_, DBE_wa_, and the proportion of the C2 component in fluorescence spectra (Table S3). The detected lipids, proteins, carbohydrates, and condensed aromatic molecules of DA were similar to those reported in the previous literature [3], and the lignin and tannin levels were slightly higher than those reported in the literature (Table S10). For the ADOM, due to the high proportion of lipids, the majority of proteins and carbohydrates originated from microbial metabolism, which are relatively easy to be degraded by microorganisms [15,55]. For MDOM, a higher percentage of aromatic structural compounds (e.g., lignin, tannins, and condensed aromatic molecules) made them resistant to microbial degradation [15] and allowed for a longer time in aquatic environments compared to ADOM [56]. Interestingly, there were significantly more condensed aromatic molecules in SRFA compared to ADOM, and ADOM also contained noticeably more lipids (Table S9). We proposed that lipids and condensed aromatic molecules are endogenous and exogenous characteristic DOM, respectively.

For SRFA, the distribution of FT-ICR MS was relatively concentrated, whereas that of Orbitrap MS was more dispersed (Figure S4; Table S11). The specific dominance of condensed aromatic molecules was reduced and replaced by more lipids, proteins, and carbohydrates [57,58]. Therefore, the accuracy of determining the source of DOM by FT-ICR MS was higher than that by Orbitrap MS. For example, the proportion of tannins identified by Orbitrap MS (3.9–16%) was higher than that by FT-ICR MS (0.8–13%). Regarding the proportion of carbohydrates, there was a sharp contrast between the results of Orbitrap MS (3–7%) and FT-ICR MS (0.3–4%). There is evidence that Orbitrap MS is more appropriate for analyzing the large amount of proteins and lipids produced by phytoplankton in the positive ion mode [40]. In this study, Orbitrap MS seems suitable for the ionization of compounds with glycosyl structures, like tannins. Nevertheless, considering the prevalence of lignin, tannins, and condensed aromatic molecules in DOM, it is usually recommended to follow the negative ion mode for detection [34]. In particular, compared to the positive ion mode in MS, the negative ion mode can detect more compounds [34].

On the whole, the CHO compounds were the main component and dominated by lignin in the VK plots of all DOM samples (Figure 3). CHON compounds exhibited low H/C and lower saturation, while CHOS and CHOP compounds were prone to high H/C and higher saturation [59]. The concentration of CHON in SRFA was predominantly concentrated in the lignin region. In contrast, the CHON in MDOM and ADOM was more evenly distributed in lipid, protein, and lignin regions. CHOS was mainly focused in the protein region of algae and HV and in the lipid and protein regions of WH and MV, respectively. Previous studies have shown that CHOS is abundant in DOM samples of Microcystis aeruginosa [60]. Moreover, it was more prevalent in macrophytes [40]. CHOP from algae and HV was concentrated in lipid regions and classified as phospholipids. Except for lipids, the CHOP of WH and MV was also distributed in protein and lignin regions. In addition, algae contained numerous CHONS and CHOSP molecules, which were also scattered in similar compound regions. Conversely, SRFA contained fewer S- and P-related species compounds, which were dispersed in regions of tannins and condensed aromatic molecules.

The Orbitrap MS results (Figure S4) indicated that CHOP exhibited the highest concentration in the protein region, which may be pointed out as phosphoproteins. DOM was also distributed in the lipid, carbohydrate, and lignin regions. In the literature [61], the MS results indicate that there is a single P atom in DOP. Phosphate esters (tri-, di-, and mono-esters) are a common form of organophosphorus [57], and various types of nucleotides also contain P [58]. P atoms may exist in the aromatic structures [62]. More DOP compounds are generated in the negative ion mode than positive ion mode [63].

### 2.4. Comparative Analysis Between FT-ICR MS and Orbitrap MS

**Figure 5:**
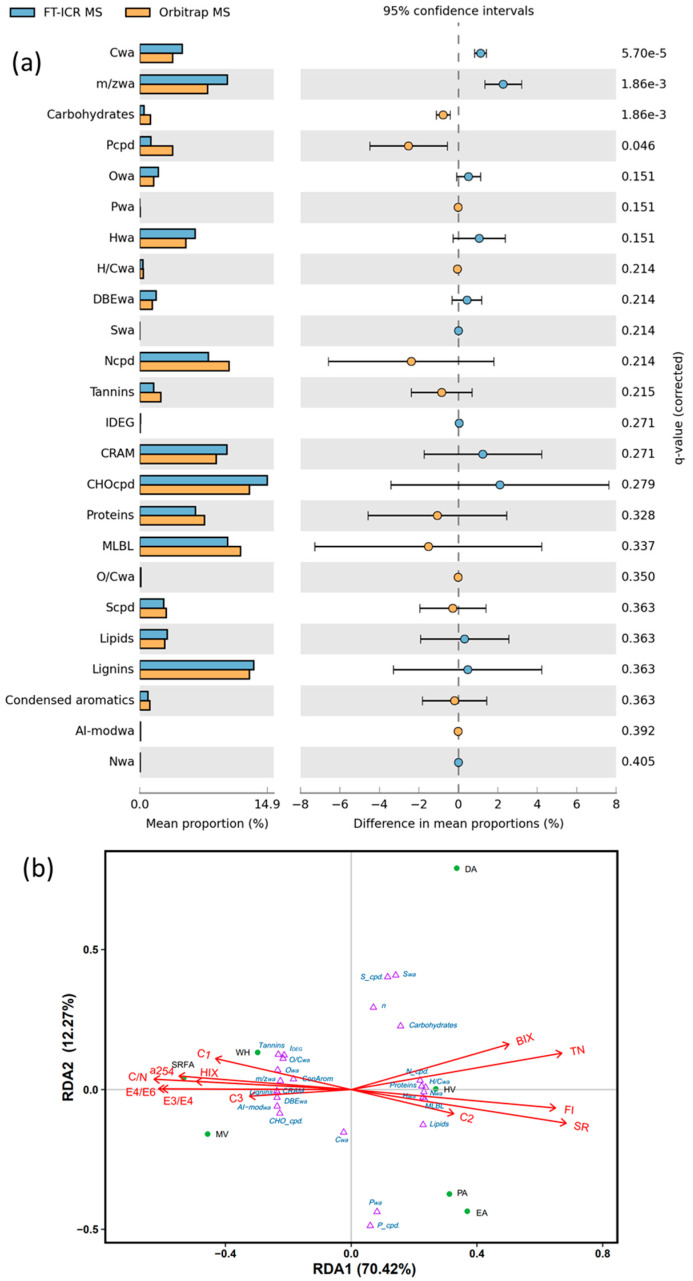
(**a**) Differential analysis of multiple parameters from FT-ICR MS and Orbitrap MS. (**b**) Multivariate analysis of compounds and drivers using RDA. In Figure 5b, ordinations were based on Bray–Curtis dissimilarities of 25 variables (purple triangle) derived from FT-ICR MS molecular composition. Optical indices were fit to the same ordination. The green center represents the sample.

Among the 24 parameters adopted in this study, there was no significant difference between FT-ICR MS and Orbitrap MS regarding 20 parameters (Figure 5a), suggesting that the DOM molecular properties detected by the two kinds of MS are highly similar. Significant (*p* < 0.05) differences among the two MS were observed in C_wa_, *m*/*z*_wa_, carbohydrates and P cpd. Specifically, the *m*/*z* of Orbitrap MS shifted toward lower molecular weights than that of FT-ICR MS and had a stronger response to heteroatom P. Moreover, Orbitrap MS can detect a higher proportion of carbohydrate and tannin compounds, making it more suitable for the ionization of compounds with glycosyl structures. In contrast, the response of FT-ICR MS to CHO-like compounds is more sensitive than that of Orbitrap MS [33]. We propose that the ion source parameters of the mass spectrometer are one of the key factors influencing DOM analysis.

To further compare the differences in DOM characteristics from different aquatic environments, a multivariate analysis of the molecular composition was carried out using redundancy analysis (RDA) (Figure 5b). The results demonstrated that the first dimension (RDA1) accounted for 70.4% of the variance in these samples. The SRFA, WH, and MV exhibiting larger *m*/*z*, aromaticity, and oxygen content (i.e., large *m*/*z*_wa_, DBE_wa_, AI-mod_wa_, O_wa_, O/C_wa_) were distributed in the negative region of the RDA1, whereas algae and HV with more aliphatic (i.e., large H_wa_ and H/C_wa_) were distributed in the positive region of RDA1. Previous studies have concluded that autochthonous sources of DOM, such as algae, have low O-content, rich H-content, low AI-mod in their molecular formulas, and more proteins and lipids [8,64]. In contrast, the molecular formula of negative RDA1 with opposite characteristics may reflect terrestrial DOM inputs, such as lignin and tannin compounds [8].

Furthermore, a_254_, HIX, E3/E4, E4/E6, C/N, and fluorescent fractions C1 and C3 showed a high degree of covariation with the three samples of SRFA, WH, and MV containing high proportions of lignin, tannin, and condensed aromatic molecules. Conversely, BIX, FI, SR, TN, and fluorescence component C2 were positively correlated with algal samples dominated by endogenous DOM inputs of lower aromaticity and RDA1. Admittedly, RDA1 provides a comprehensive explanation for the convergence of DOM from aromatic to aliphatic in aquatic environments [30]. The second dimension (RDA2) explained 12.3% of the variance in the samples, which was attributed to the content of S and P heteroatoms. A significant dispersion was observed between S-related parameters (S cpd. and S_wa_ for RDA2 > 0) and P-related parameters (P cpd. and P_wa_ for RDA2 < 0). The parameters S cpd., S_wa_, n, carbohydrate, protein, and DA achieved high scores and were distributed in the same interval (RDA1 > 0, RDA2 > 0), suggesting that S atoms originated more from carbohydrate and protein structures and were also more readily detected by FT-ICR MS. Conversely, parameters P cpd., P_wa_, lipid, PA, and EA exhibited comparable scores and were distributed within the same interval (RDA1 > 0, RDA2 < 0), indicating that P atoms were more likely to originate from lipid structures. The DOM of algae contained a greater abundance of aliphatic compounds comprising N, S, and P atoms, and these heteroatoms further enhanced the microbial activity and the rate of DOM degradation [55]. Overall, this study indicated the effectiveness of combining simple and efficient spectral techniques in conjunction with high-cost and high resolution MS techniques, facilitating the comprehensive evaluation of DOM composition in aquatic ecosystems.

### 2.5. Characterization of LMW-DOM by Orbitrap MS Analysis

As demonstrated in Figure S1, substantial ionic peaks were identified in the DOM of all samples within the range of *m*/*z* < 200 Da, accounting for 9–13% of the Bulk-DOM (Table S12). This indicated that small molecules may have potential environmental effects on the aquatic environment. This assertion is also supported by prior characterization of SRFA dialysate with 100–500 Da interception of molecular weight cut off by Orbitrap MS [9], comprising lignin and some compounds derived from macrophytes [17]. A similar observation was made in the Suwannee River DOM (2R101N) with *m*/*z* < 200 Da fraction in the IHSS, which occupied 10.7% of Bulk-DOM [36]. Furthermore, the presence of small-molecular-weight ionic peaks occurred in ADOM and MDOM (Table S12). Previous studies have employed Orbitrap and FT-ICR MS to characterize DOM in lakes, confirming that the fractions with *m*/*z* < 200 were 11.4% and 7.7%, respectively, with the vast majority originating from fresh DOM produced by algae [36]. Higher proportions of LMW-DOM (<350 Da) have been documented in surface water, ranging from 54% to 74% [65]. The possible origins of these fractions include the inputs of terrestrial material or decomposing products from algae and macrophytes in the aquatic environment.

The O/C and H/C of LMW-DOM shifted from dispersed to clustered distribution (Figures S5 and S6), indicating high similarity in all DOM samples. Compared to Bulk-DOM, the LMW-DOM in ADOM had higher oxygen content, lower saturation, and more aromatic, while the LMW-DOM in MDOM and SRFA showed lower oxygen content, higher saturation, and more aliphatic. This finding was corroborated by the molecular parameters of the same LMW-DOM (Table S13). The DBE_wa_ and AI-mod_wa_ of LMW-DOM from SRFA and MDOM were larger than those of ADOM, suggesting a greater degree of unsaturation and aromaticity. Unlike Bulk-DOM, the NOSC of LMW-DOM was negative (<0) for all samples, indicating that the small molecules were in the reduced state and less stable. Besides, LMW-DOM had less CRAM% relative to Bulk-DOM, which further consolidates the higher activity of LMW-DOM.

The molecular composition of LMW-DOM was dominated by CHO and CHON, which were distributed in seven discrete regions of compounds. Notably, the proportion of N-containing compounds in LMW-DOM (37–53%) was higher than that of Bulk-DOM (Table S13). For LMW-DOM, the order of biochemical reactivity was as follows: dissolved organic nitrogen (DON) ≥ dissolved organic carbon (DOC) > dissolved organic phosphorus (DOP) [11]. Thus, the lability of LMW-DOM was greater than that of Bulk-DOM due to its higher content of N atoms. The trophication degree of lakes has a profound impact on ADOM. The higher the trophic level (DA > PA > EA), the more CHON (210 > 171 > 141) and total N-containing compounds (260 > 277 > 248). Eutrophication has been proven to affect the production of N-containing compounds, so the heteroatom LMW-DOM is closely associated with microbial utilization and nutrient cycling in aquatic ecosystems [17,66].

The prevalence of LMW-DOM carrying S and P heteroatoms in algae was considerably higher than that in macrophytes (Table S12). The S-containing LMW-DOM predominantly existed in the protein, carbohydrate, and lignin, as well as the tannin and condensed aromatic molecule regions from DA and SRFA. The LMW-DOM in DA contained the most S-containing compounds. Specifically, CHOS mainly existed in the form of O_2_S, O_3_S, and O_4_S, while CHONS was composed of NO_2_S, NO_3_S, and NO_4_S (Table S14). Notably, the proportion of S-containing compounds in the LMW-DOM was comparable to that in Bulk-DOM (Table S4). For the P-containing compounds in LMW-DOM, the O_4_P and O_5_P were the predominant components in CHOP (Table S15), accompanied by traces of CHONP and CHOSP. The P-containing LMW-DOM were primarily found in the carbohydrates, lignin, and tannins of algae and macrophytes (Table S16). Besides, the proportion of P-containing compounds in LMW-DOM was lower than that in Bulk-DOM (Tables S8 and S12).

To obtain more information on DOM, we matched possible structures through an online database using the chemical formulas of DOM (Tables S17 and S18). Further, according to the molecular structural formulas of DOM, we demonstrated three common DOM formation reactions, including dehydration, decarbonation, and demethylation (Figure S7). For instance, the representative glucopyranose undergoes dehydration reactions at the hydroxyl groups at 1 and 6 sites to obtain levoglucosan (Figure S7a), and adipic acid with terminal carboxyl groups is converted into valeric acid through decarbonization (Figure S7b). The (E)-ferulic acid with a typical lignin structure undergoes a demethylation reaction to form caffeic acid (Figure S7c).

### 2.6. Environmental Implications

In recent decades, the proliferation of algae and macrophytes worldwide is the result of water quality deterioration or eutrophication caused by human activities. These blooms have led to an increase in heteroatom DOM and LMW-DOM levels, which play a crucial role in the supply of organic matter pools in lake ecosystems. The source and molecular composition of these DOM determine their biogeochemical processes in aquatic ecosystems and play a non-negligible role in the global ecological cycle. Due to the chemical complexity of DOM, it is challenging to fully elucidate its molecular composition and properties using a single characterization technique. The integration of spectroscopic methods with mass spectrometric techniques can effectively validate the accuracy and reliability of the obtained results.

The source resolution of lake DOM is determined by the characteristic DOM components identified through high-resolution mass spectrometry. Lipids are representative endogenous DOM components. In contrast, condensed aromatic molecules are indicative of exogenous DOM sources. The ecological effect assessment of lake DOM is primarily based on the evaluation of LMW-DOM and DOP. We found that the percentage of compounds with molecular weight < 200 Da identified by Orbitrap MS was approximately 10% of the Bulk-DOM. Previous studies have shown that LMW-DOM is usually biologically labile [67]. Bulk-DOM was predominantly composed of CHO, while LMW-DOM consisted of CHO and CHON. In comparison to Bulk-DOM, LMW-DOM exhibited reduced stability and augmented activity. Consequently, LMW-DOM in eutrophic lakes might result in the accumulation of more soluble and decomposable reactive substances, thereby accelerating the mineralization of organic matter in eutrophic lakes. It was, therefore, hypothesized that eutrophication processes and algal bloom outbreaks led to changes in heteroatom DOM and LMW-DOM compositions in lakes. These changes, in turn, affect the biogeochemical cycling (e.g., degradation and deposition) and pollutant behaviors (e.g., transport and transformation) of organic matter. For example, DOP is susceptible to photodegradation reactions. In summary, heteroatom DOM and LMW-DOM merit heightened consideration as a significant reservoir of organic matter in eutrophic environments. Future research should focus on investigating the mechanisms of endogenous organic matter reservoirs in eutrophic lakes within larger ecosystem frameworks and examining the environmental behavior of organic matter at the molecular level.

## 3. Materials and Methods

### 3.1. Sample Collection and Preparation

To examine ADOM, algae were independently sampled from Dianchi Lake (102°42′36″ E; 24°48′55″ N), Erhai Lake (100°09′51″ E, 25°53′42″ N), and Puding Reservoir (105°49′, 26°22′) in 2022. The corresponding trophic levels in these water bodies are eutrophic, mesotrophic, and meso-eutrophic transition states, respectively. Considering the different aquatic environments, these algae samples were named EA, PA, and DA [8]. At each site, we collected algae with phytoplankton nets at 0.5 m underwater. After freeze-drying, these algae were ground to pass through a 100-mesh screen and then stored at −20 °C.

To investigate MDOM, we collected one floating plant and two submerged plants from the Puding Karst Ecosystem Observation and Research Station, Chinese Academy of Sciences (105°42′20″~105°46′11″ E; 26°15′41″~26°21′44″ N) in September 2020. That is WH, MV, and HV, respectively. After cleaning, the three macrophytes were placed in sterile bags and transferred to the laboratory. After freeze-drying, these samples were ground to pass through a 100-mesh screen and then stored at −20 °C.

In order to increase the comparative study of terrigenous organic matter, we intentionally bought the standard sample of SRFA from the International Humic Substances Society (IHSS). The SRFA solution was first prepared to a stock concentration of 1000 mg C·L^−1^ and then diluted to suit different experiments.

### 3.2. Organic Element Analysis

A total of 10–20 mg of macrophyte or algal powder was weighed into a tin boat. Based on the high-temperature combustion method, the contents of TOC and TN were determined using a German Elementar element analyzer (Vario macro cube) (Elementar, Langenselbold, Germany). The C/N molar ratio was calculated according to the relative atomic mass. The organic element analysis data of the SRFA were referred to the IHSS.

### 3.3. DOM Extraction

The sample powder (0.3 g macrophyte or 50 mg algae) was added to 30 mL Milli-Q water and oscillated in a shaker at 25 °C for 18 h without light. After centrifugation, the supernatant was passed through a 0.45 µm glass fiber filter membrane to obtain extracted DOM and then preserved in brown glass vials for DOC analysis, UV-Vis, EEMs, and MS.

### 3.4. Optical Spectroscopy Analysis

DOC concentrations were determined by a total organic carbon analyzer (Aurora 1030W, OI Analytical, College Station, TX, USA) in parallel, with an analytical error of less than ±1.5%. The UV-Vis and EEMs of the DOM extract were synchronously measured using a fluorescence spectrometer (Aqualog-UV-800-C, Horiba, Kyoto, Japan). The scan range for Ex was 240–800 nm at an interval of 5 nm, while Em was 240–800 nm at an interval wavelength 1 nm. The scanning integration time was 0.3 s, and the scanning speed was 1200 nm·min^−1^. The inner filter effect, Rayleigh masking, and Raman normalized 3D correction were performed using the Aqualog^®^ system [68]. The software (Matlab R2014b, Mathworks, Natick, MA, USA) was used to perform parallel factor analysis (PARAFAC) on the EEM data to determine the fluorescence components by residual analysis [69]. Meanwhile, the absorption coefficient, absorbance ratio, and spectral slope ratio of the UV-VIS absorption spectrum were also calculated. The detailed calculation of EEMs and UV-Vis parameters is provided in the Supplementary Materials, including a_254_, a_300_, E2/E3, E3/E4, E4/E6, spectral S_R_, FI, HIX, and BIX, which are described in the Supplementary Materials.

### 3.5. Mass Spectrometry Analysis

The DOM components in the extracts of algae and macrophytes samples were pre-enriched by a solid phase extraction (SPE) column for molecular characterization. Briefly, the SPE column (Agilent Bond Elute PPL, 500 mg/6 mL) was first pre-activated with methanol (LC-MS grade) and with pure HCl until pH 2.0. After that, the SPE column was washed with three column volumes of acidified ultra-pure water to completely eliminate the salt. Then, the column was dried with nitrogen gas and eluted with 6 mL of methanol, and the eluent was collected in a brown glass bottle. Aliquots eluents (0.3 mL) were N2 dried and then diluted by 30 mL Milli-Q water to calculate the extraction efficiency. The remaining eluents were N2 dried and re-dissolved in methanol for the detection of FT-ICR MS and Orbitrap MS.

The molecular composition of DOM in the above methanol eluents was analyzed by 9.4 T FT-ICR MS and Orbitrap MS. The samples were directly injected into an electrospray ionization (ESI) unit in negative ion mode for two kinds of MS analysis. Molecular formulas containing the elements C, H, O, N, P, and S were assigned using routine software (CUPBPEC-HRMS-Viewer V1.6) [17,70,71]. The typical element constraints (C_c_H_h_N_n_O_o_S_s_P_p_, c < 50, h < 80, o < 30, n < 4, s < 2, p < 1) and heuristic rules were integrated to eliminate cases where multiple formulae were assigned to the same *m*/*z*.

The molecular compounds in the VK diagram were divided into different compounds based on the H/C and O/C ratios: (1) lipid-like (1.5 ≤ H/C ≤ 2.0, 0 ≤ O/C ≤ 0.3), (2) protein/peptide-like (1.5 ≤ H/C ≤ 2.2, 0.3 < O/C ≤ 0.67), (3) carbohydrate-like (1.5 ≤ H/C ≤ 2.4, 0.67 < O/C ≤ 1.2), (4) unsaturated hydrocarbon-like (0.7 ≤ H/C < 1.5, 0 ≤ O/C ≤ 0.1), (5) lignin-like (0.7 ≤ H/C < 1.5, 0.1 < O/C ≤ 0.67), (6) tannin-like (0.5 ≤ H/C < 1.5, 0.67 < O/C ≤ 1.2), and (7) condensed aromatic molecules (0.2 ≤ H/C < 0.7, 0 ≤ O/C ≤ 0.67) [72]. More details of MS indices and calculation formulas are provided in the Supplementary Materials.

### 3.6. Statistical Analysis

Significant differences between FT-ICR MS and Orbitrap MS regarding multiple parameters were analyzed using STAMP2.1.3 software [30]. RDA of FT-ICR-MS data and optical parameters was performed in R V4.2.1 using the vegan package. Input parameters for sorting included most properties (i.e., TN, C/N, a_254_, E3/E4, E4/E6, SR, C1–C3%, FI, HIX, and BIX); the total number of assigned formulas (n); the percentage of assigned formulas that identified CHO-, N-, S- and P-containing compounds (CHO cpd, N cpd, S cpd, P cpd); the intensity-weighted average parameters *m*/*z*_wa_, H/C_wa_, O/C_wa_, DBE_wa_, AImod_wa_, etc.; and relative abundances of compound classes, including lipids, proteins, carbohydrates, lignin, tannins, and condensed aromatic structures (ConArom). Additionally, ordinations were conducted based on Bray–Curtis dissimilarities of variables derived from FT-ICR-MS molecular composition, as well as optical parameters.

## 4. Conclusions

Overall, this study investigated the Bulk-DOM composition of MDOM and ADOM from contrasting plateau lakes using optical spectroscopy and high-resolution mass spectrometry. We found that ADOM exhibited the characteristics of endogenous DOM, while the MDOM showed the characteristics of both endogenous and terrigenous DOM. ADOM was characterized as having a relatively higher content of N, S, and P heteroatoms and a larger proportion of MLBL, abundant proteins, carbohydrates, and lipids than MDOM. ADOM was more aliphatic and biodegradable. Orbitrap MS was superior to FT-ICR MS in terms of its ability to characterize P-containing molecules. There were significant (*p* < 0.05) differences between FT-ICR and Orbitrap MS in C_wa_, *m*/*z*_wa_, carbohydrates, and P-containing compounds. Intriguingly, LMW-DOM (<200 Da) accounted for approximately 10% of Bulk-DOM and was dominated by CHO and CHON. Compared to Bulk-DOM, the LMW-DOM in ADOM had higher oxygen content and lower saturation and was more aromatic, while the LMW-DOM in MDOM and SRFA showed lower oxygen content and higher saturation and was more aliphatic. The LMW-DOM was more active than Bulk-DOM because of the reduced state and a higher percentage of N-containing compounds. The P-containing LMW-DOM were primarily found in the carbohydrates, lignin, and tannins of algae and macrophytes. These findings provide new perspectives into the composition, origin, and fate of MDOM and ADOM in lakes under different trophic states, which may further deepen our understanding of algal blooms in eutrophic plateau lakes.

## Figures and Tables

**Table 1:** Intensity-weighted mean molecular parameters of different samples derived from assigned molecular formulae by FT-ICR MS ^a^.

Sample	*m*/*z*_wa_	*n*	C_wa_	H_wa_	O_wa_	N_wa_	S_wa_	P_wa_	H/C_wa_	O/C_wa_	DBE_wa_	AI-Mod_wa_	NOSC_wa_	I_DEG_	CRAM (%)	MLBL (%)	CHO Cpd. (%)	N Cpd. (%)	S Cpd. (%)	P Cpd. (%)
**WH**	395.94	3177	17.35	18.65	10.45	0.03	0.03	0.01	1.14	0.61	8.54	0.31	0.15	0.40	42.78	29.78	78.06	12.78	6.70	2.90
**MV**	414.33	1514	18.89	17.95	10.44	0.02	0.03	0.04	1.02	0.56	10.44	0.40	0.16	0.84	52.18	19.75	77.74	9.51	8.98	5.02
**HV**	376.32	6831	19.45	30.29	6.57	0.36	0.05	0.01	1.61	0.35	4.99	0.12	−0.80	0.10	34.36	52.72	41.17	49.90	7.28	2.77
**EA**	358.82	3949	18.77	29.26	5.96	0.33	0.06	0.06	1.62	0.32	4.84	0.13	−0.87	0.07	29.41	60.72	44.87	40.59	9.57	9.01
**PA**	375.67	5157	19.53	29.24	6.46	0.34	0.06	0.06	1.55	0.34	5.61	0.16	−0.77	0.06	30.91	55.87	45.32	41.17	11.19	9.04
**DA**	372.20	7817	18.48	28.39	6.92	0.33	0.19	0.01	1.59	0.39	4.96	0.11	−0.68	0.34	30.74	54.27	36.04	43.26	25.06	1.62
**SRFA**	437.41	7759	20.52	20.02	10.57	0.05	0.02	0.01	1.03	0.52	11.04	0.41	0.07	0.68	50.64	5.21	73.00	20.08	6.64	4.55

^a^ Intensity-weighted average values are displayed for mass-to-charge ratio (*m*/*z*_wa_), number of carbon (C_wa_), hydrogen (H_wa_), oxygen (O_wa_), nitrogen (N_wa_), sulfur (S_wa_), and phosphorus atoms (P_wa_), hydrogen-to-carbon ratio (H/C_wa_), oxygen-to-carbon ratio (O/C_wa_), modified aromaticity index (AI-modwa), nominal oxidation state of carbon (NOSC_wa_), double bond equivalent (DBE_wa_), and degradation index of DOM (IDEG). Abbreviations: MLBL, natural OM behaving as a labile substance value; CRAM, carboxyl-rich alicyclic molecules value; and n, number of identified molecules. The CHO cpd. (%) represents the percentage of compounds containing only C, H, and O; the N, S, P cpd. (%) represent the percentage of nitrogenous compounds, sulfur compounds, and phosphorous compounds, respectively.

## Data Availability

No new data were created or analyzed in this study. Data sharing is not applicable to this article.

## Supplementary Materials

The following supporting information can be downloaded at https://www.mdpi.com/article/10.3390/molecules30173510/s1, Figure S1: Mass spectrum peak pattern of macrophyte and algae, (a), (c), (e), (g), (i), (k), (m) from FT-ICR MS, (b), (d), (f), (h), (j), (i), (n) from Orbitrap MS; Figure S2: Bubble diagram of macrophyte and algae via (a) FT-ICR MS or (b) Orbitrap MS; Figure S3: The Van Krevelen (VK) plots of SRFA characterized by (a) FT-ICR MS and (b) Orbitrap MS; Figure S4: VK maps of compounds in macrophyte, algae, and SRFA via Orbitrap MS; Figure S5: Bubble diagram of the LMW-DOM in various samples tested by Orbitrap MS; Figure S6: VK maps of LMW compounds in macrophytes, algae, and SRFA via Orbitrap MS; Figure S7. The mechanism of the DOM formation via (a) dehydration, (b) decarbonation, and (c) demethylation; Table S1: Results of elemental analysis and C/N ratios of macrophyte and algae; Table S2: The optical parameters of UV-Vis absorption spectra of the macrophyte, algae, and standard sample; Table S3: Proportions of three fluorescence components and characteristic values of fluorescence spectra from different DOM samples; Table S4: Intensity-weighted mean molecular parameters of different samples derived from assigned molecular formulae by Orbitrap MS; Table S5: Comparison of literature results on SRFA and DOM extraction from algae and macrophyte; Table S6: Distribution of O_3_S and O_5_S compounds in algae; Table S7: Composition of major subcategories of DOM in various samples analyzed by FT-ICR MS; Table S8: Composition of major subcategories of DOM in various samples analyzed by Orbitrap MS; Table S9: Relative abundance (%) of van Krevelen diagram classification from FT-ICR MS analysis of DOM from various sources; Table S10: Comparison of the classification derived from the van Krevelen diagram in the literature for DOM from algae, macrophyte, and SRFA; Table S11: Relative abundance (%) of van Krevelen diagram classification from Orbitrap MS analysis of DOM from various sources; Table S12: Composition of major subcategories of LMW-DOM in various samples analyzed by Orbitrap MS; Table S13: Intensity-weighted mean molecular parameters of LMW-DOM in various samples analyzed by Orbitrap MS; Table S14: Distribution of S-containing LMW-DOM in DA; Table S15: Distribution of P-containing LMW-DOM in macrophyte, algae, and SRFA via Orbitrap MS, where figures represent numbers; Table S16: Relative abundance (%) of van Krevelen diagram-derived classification from Orbitrap MS analysis of LMW-DOM; Table S17: The possible structures and names of DOM with high strength; Table S18: The possible structures and names of LMW-DOM with high strength.
