# Supplementary material for: Unraveling the Molecular Composition and Reactivity Differentiation of Algae- and Macrophyte-Derived Dissolved Organic Matter in Plateau Lakes: Insights from Optical Properties and High Resolution Mass Spectrometry Characterization
Source: Molecules. 2025 Aug 27;30(17):3510. doi: 10.3390/molecules30173510 (PMC12430184; doi:10.3390/molecules30173510)
Supplement: Supplementary file 1 [file molecules-30-03510-s001.zip › molecules-3789746-supplementary.pdf]

Supplementary Materials

Unraveling the molecular composition and reactivity differentiation of algae- and macrophyte-derived dissolved organic matter in plateau lakes: Insights from optical properties and high resolution mass spectrometry characterization

Qiuxing Li, Runyu Zhang, Haijun Yuan, Liying Wang, Shuxia Xu

1. Mass spectrometry analysis

The molecular composition of DOM in all above methanol eluant (i.e. DOM samples) was analyzed using a Fourier transform ion cyclotron resonance mass spectrometer (FT-ICR MS, 9.4T Apex, Bruker, Germany). The spray shielding voltage was 3.3 kV, the capillary column inlet voltage was 3.8 kV, and the capillary column end voltage was -300V. The ions accumulate in the collision pool for 1.0 s and then extracted into the ICR pool with a time of flight (TOF) of 1.2 ms. The optimized mass (Q1) of the quadrupole was 300 Da. Quality range was 200–800 Da. The samples were directly injected into an electrospray ionization (ESI) unit at 180 μL/h in negative ion mode. A total of 128 time-domain signals of 2 M word length were added (*ACS Omega* **2020**, 5, 5372-5379). Internal calibration and extraction of mass lists of FT-ICR mass spectra were performed with DataAnalysis 3.4 (Bruker, Germany). Mass peaks with signal to noise ratio (s/n) magnitude greater than 6 were exported to peak lists. Molecular formula assignments of mass lists of FT-ICR MS were performed using custom software. Also, it should be noted that all molecular formulae in this article refer to $[M - H]^-$. The normalized intensities were calculated by dividing the intensity of each peak by the sum of the intensities of all assigned molecular formulae in one broadband mass spectrum (*ACS Omega* **2020**, 5, 5372-5379).

DOM samples were also analyzed by a quadrupole-Orbitrap MS (Thermo Scientific, Q Exactive, USA), equipping with high performance liquid chromatograph (Thermo Scientific, U3000, USA). External calibration was performed prior to testing using a manufacturer-specified calibration mixture. The negative ion mode used ESI source, and the mass-to-charge ratio (m/z) ranges from 70–1050 Da at resolution of 140000. The operating parameters of the Orbitrap MS instrument were as follows: resolution, 140,000; spray voltage, 3.0 KV; capillary temperature, 350 °C; heater temperature, 300 °C; sheath gas flow rate, 40 psi; auxiliary gas flow rate, 10 arb; s-lens RF level, 50%; and AGC target, 3×10^6. The original mass spectrum data was processed by software Xcalibur Qual Browser (Thermo scientific, USA). After integrating all the scanned frame data, the mass charge ratio, peak intensity and relative intensity were extracted. The molecular formulas of the DOMs were processed *via* the software ICBM-OCEAN (version 1.0) and assigned by referring to previous studies (*Anal. Chem.* **2020**, 92, 6832-6838).

2. Parameter Calculation

2.1 Parameter Calculation of EEMs

Fluorescence index (FI) was calculated as the ratio of emission intensity at 470 nm to that of 520 nm for a fixed excitation wavelength of 370 nm (*Limnol. Oceanogr.* **2001**, 46, 38-48). Humification index (HIX) was calculated as the ratio of Em fluorescence peak area between 435–480 nm and 300–345 nm when Ex = 250 nm (*Limnol. Oceanogr.* **2010**, 55, 2645-2659). Biological index (BIX) was calculated as the ratio of fluorescence intensity of Em at 380 nm and 430 nm when Ex = 310 nm (*Org. Geochem.* **2009**, 40, 706-719).

2.2 Parameter Calculation of UV-Vis

The a_{254} and a_{300} were absorbance at 254 nm and 300 nm, respectively. The absorbance ratios at 250 to 365 nm (E2/E3), 300 to 400 nm (E3/E4) and 465 to 665 nm (E4/E6) were calculated following the definitions in previous literature (*Water Res.* **2021**, 207, 117833; *J. Environ. Sci.* **2009**, 21, 581-588). The spectral slope ratio (S_R) was defined as the ratio of the spectral slopes S of the shorter (275–295 nm) to the longer (350–400 nm) wavelength ranges (*Limnol. Oceanogr.* **2008**, 53, 955-969).

2.3 Parameter Calculation of Mass Spectrum Data

For compounds with the formula $C_cH_hN_nO_oS_sP_p$, the unsaturation of a single molecule was usually described as double bond equivalent (DBE) (*Environ. Sci. Technol.* **2024**, 22, 100470). DBE was numerically equal to the sum of the number of molecular rings and the number of double bonds, and the calculation formula of DBE was shown as follows:

$$\mathrm{DBE} = \mathrm{c} - \frac{h}{2} + \frac{n}{2} + 1$$

After obtaining the chemical parameters of individual molecules based on their elemental composition, the weighted average parameters of these compounds could be calculated based on the relative content (peak strength) of these compounds. For a molecular parameter p, the weighted average was calculated as follows:

$$\mathrm{p}_{wa} = \frac{\sum p_i \cdot Intensity}{\sum Intensity}$$

The AI (Aromatic Index) parameter was proposed to reflect the aromaticity of a compound to estimate the fraction of aromatic and concentrated aromatic groups. Then, according to the practical application of molecular composition, a modified aromaticity index (AI_{mod}) was proposed (*Rapid Commun. Mass Spectrom.* **2006**, 20, 926-932). The calculation formula of AI_{mod} was shown follows:

$$AI_{mod} = \frac{1 + c - 0.5o - s - 0.5h}{c - 0.5o - s - n - p}$$

The degradation index of DOM (I_{DEG}) was used to reflect the hard degradation of DOM. The parameter calculation was based on 10 representative DOM molecules that were widely presented in environmental samples and were significantly correlated with the ^{14}C isotope abundance of the samples. Five of the negative correlating compounds were $C_{21}H_{26}O_{11}$, $C_{17}H_{20}O_9$, $C_{19}H_{22}O_{10}$, $C_{20}H_{22}O_{10}$ and $C_{20}H_{24}O_{11}$, while five of the positive correlating compounds were $C_{13}H_{18}O_7$, $C_{14}H_{20}O_7$, $C_{15}H_{20}O_7$, $C_{15}H_{20}O_8$ and $C_{16}H_{24}O_8$, respectively (*Biogeosciences* **2012**, 9, 1935-1955; *Environ. Sci. Technol.* **2023**, 57, 21145-21155; *Environ. Sci. Technol.* **2023**, 57, 10415-10425). The value of I_{DEG} range was from 0 to 1. The higher of the I_{DEG} value meant the greater of degradation degree, the greater of degradation difficulty, the older of DOM age, and the more stable of DOM. The formula for calculating I_{DEG} was as follows:

$$I_{DEG} = \frac{\sum magnitudes\ (NEG_{Ideg})}{\sum magnitudes\ (NEG_{Ideg} + POS_{Ideg})}$$

The normal oxidation state of carbon (NOSC) was used to reflect the redox potential of a given formula (*Environ. Sci. Technol.* **2024**, 22, 100470).

NOSC > 0 indicated that the compound was in the oxidation state, NOSC < 0 indicated that the compound was in the reduction state, and a value of 0 indicated that the compound was in the neutral state. The formula for calculating NOSC was as follows:

$$\mathrm{NOSC} = 4 - \frac{4 \times c + h - 2 \times o - 3 \times n + 5 \times p - 2 \times s}{c}$$

The carboxyl-rich alicyclic molecules (CRAM) was used to represent refractory dissolved organic matter (RDOM), which was a complex mixture of carboxylate and condensed aliphatic ring structure (*Water Res.* **2022**, 224, 119073). CRAM was usually associated with refractory compounds containing CHO, CHON, and a small number of CHOS molecules.

CRAM% was calculated by dividing the number of molecules DBE/C = 0.3–0.68, DBE/H = 0.2–0.95, and DBE/O = 0.77–1.75 by the total number of molecular formulas (*Cosmochim. Acta* **2006**, 70, 2990-3010).

The molecular lability boundary index (MLB) were used the H/C = 1.5 as the boundary. When H/C ≥ 1.5, the natural organic matter behaved as a labile substance (MLBL). The MLBL% obtained from dividing the number of molecular formulas of H/C ≥ 1.5 by the total number of molecular formulas. The MLBL% could be used to evaluate the instability of organic compounds (*Environ. Sci. Technol.* **2024**, 22, 100470; *Rapid Commun. Mass Spectrom.* **2015**, 29, 2385-2401). A larger MLBL% value represented greater molecular instability of DOM.

3. Figures and Tables

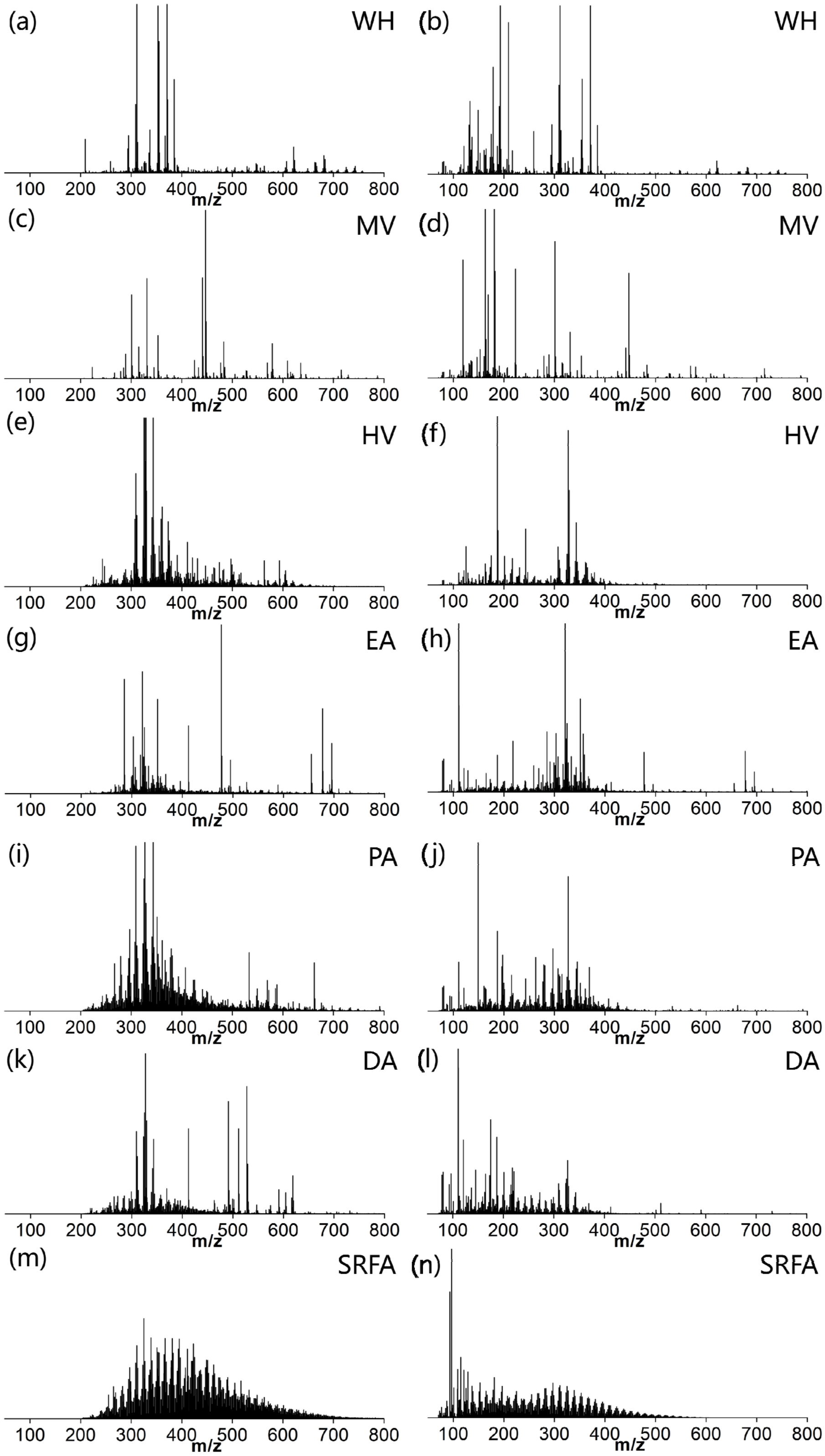

Figure S1. Mass spectrum peak pattern of macrophyte and algae, (a), (c), (e), (g), (i), (k), (m) from FT-ICR MS, (b), (d), (f), (h), (j), (i), (n) from Orbitrap MS.

The solid phase extraction (SPE) rates of macrophytes ranged from 26 to 68%, while those of algae ranged from 39 to 54%. These rates are comparable to the previously reported experimental results (20–70%) (*Sci. Total Environ.* **2020**, 703, 134764; *Environ. Sci. Technol.* **2018**, 52, 6771-6779). The loss of a small portion of DOM during SPE is primarily attributed to two factors. Firstly, some highly polar components cannot be effectively adsorbed by polyphenylene (PPL) materials. Secondly, the weakly polar components adsorbed by the PPL materials cannot be completely eluted by methanol (*Environ. Pollut.* **2022**, 312, 119992).

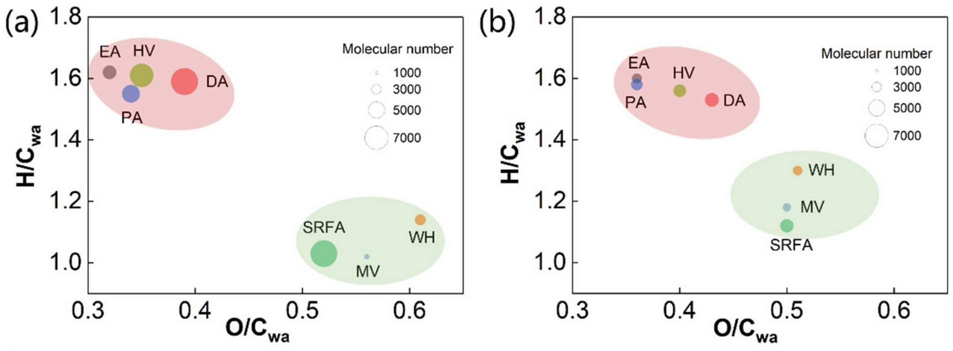

Figure S2. Bubble diagram of macrophyte and algae *via* (a) FT-ICR MS or (b) Orbitrap MS.

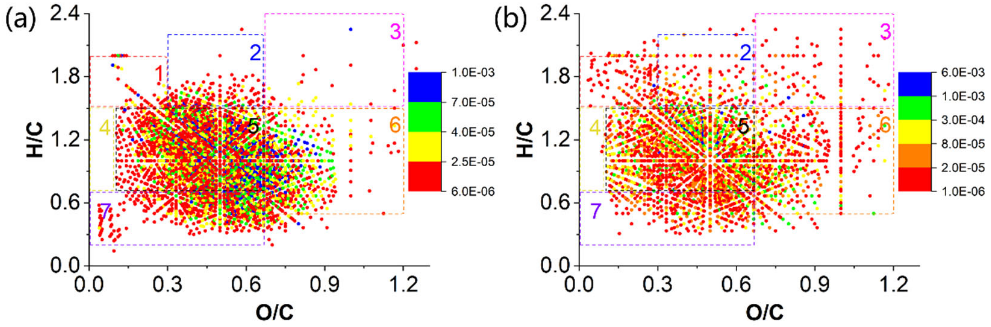

Figure S3. The Van Krevelen (VK) plots of SRFA characterized by (a) FT-ICR MS and (b) Orbitrap MS. The normalized peak intensity is indicated by color. The numbers in the figure represent (1) lipids, (2) proteins (including peptides), (3) carbohydrates, (4) unsaturated hydrocarbons, (5) lignins, (6) tannins, and (7) condensed aromatic molecules.

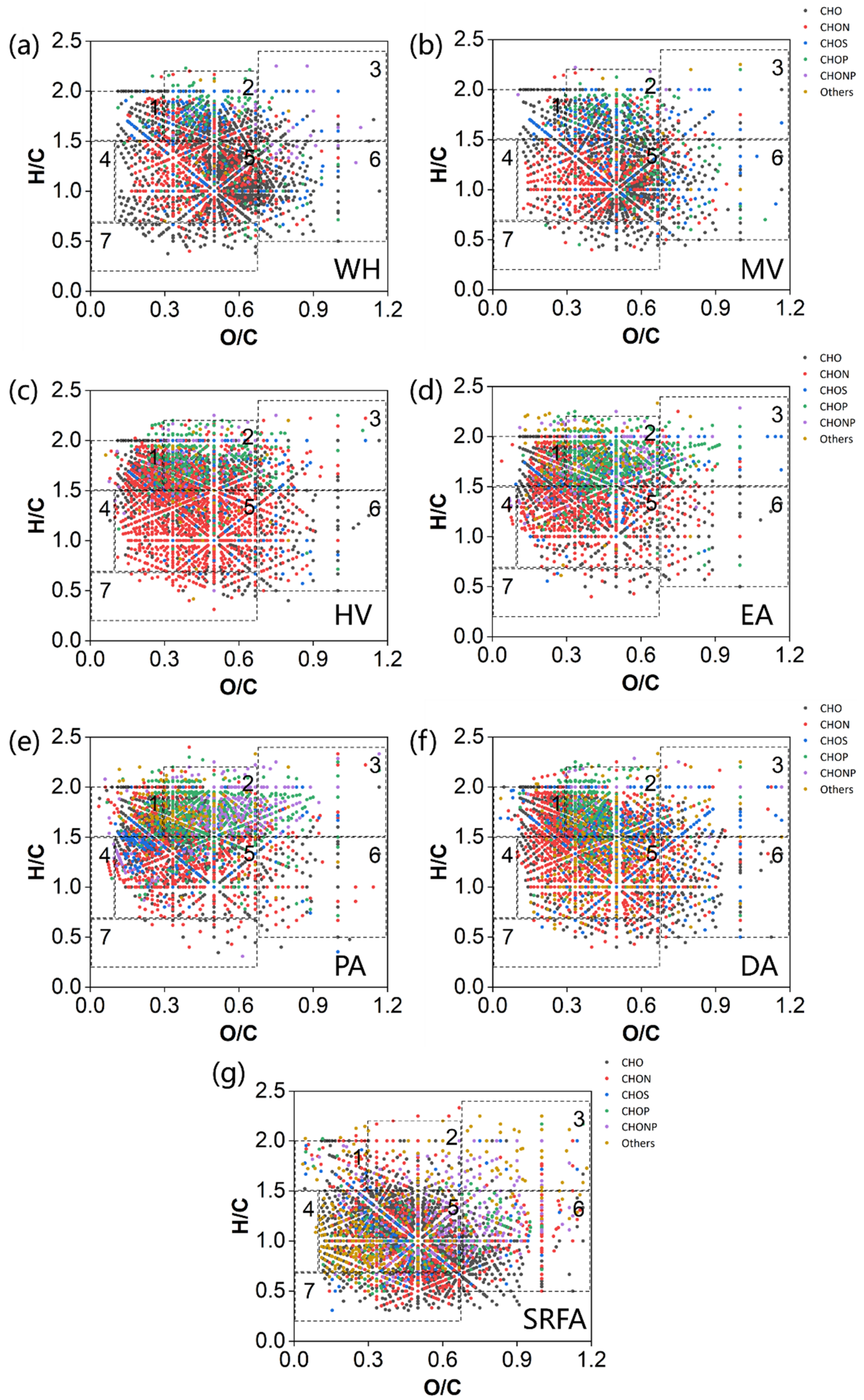

Figure S4. VK maps of compounds in macrophyte, algae, and SRFA *via* Orbitrap MS. (a) WH, (b) MV, (c) HV, (d) EA, (e) PA, (f) DA, and (g) SRFA. Compound classification marks (1–7) as above.

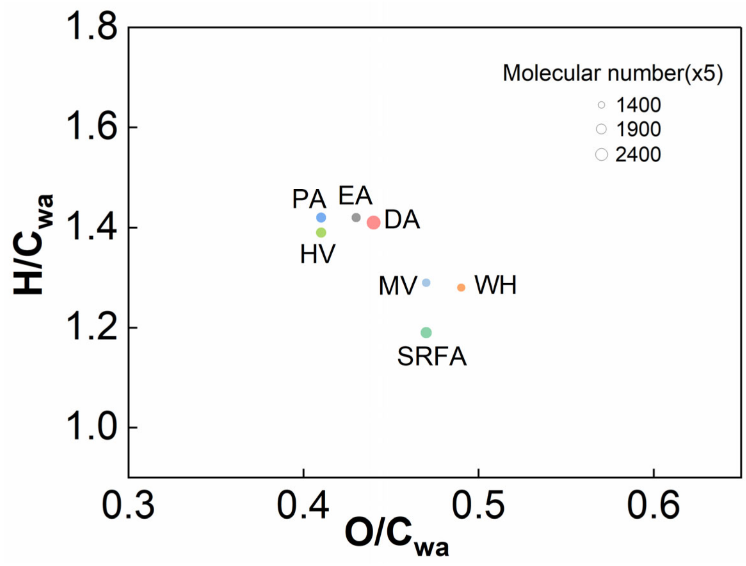

Figure S5. Bubble diagram of the LMW-DOM in various samples tested by Orbitrap MS.

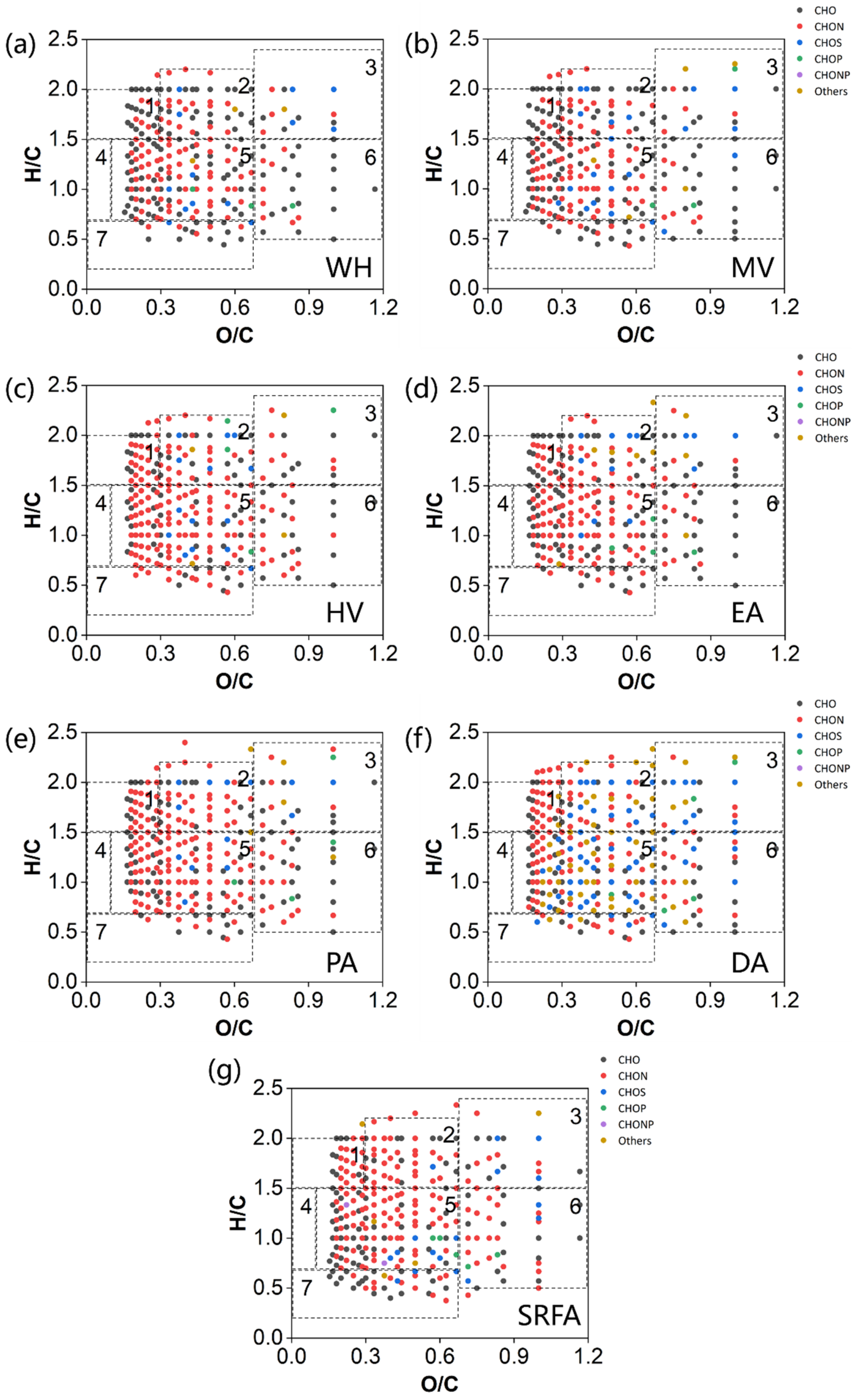

Figure S6. VK maps of LMW compounds in macrophytes, algae, and SRFA *via* Orbitrap MS. (a) WH, (b) MV, (c) HV, (d) EA, (e) PA, (f) DA, and (g) SRFA. Compound classification marks (1–7) as above.

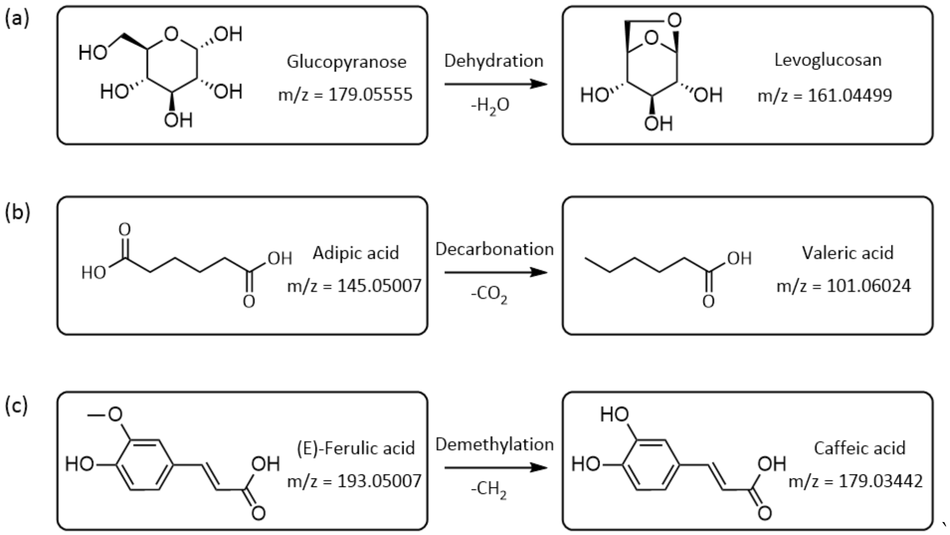

Figure S7. The mechanism of the DOM formation via (a) dehydration, (b) decarbonation and (c) demethylation.

Table S1. Results of elemental analysis and C/N ratios of macrophyte and algae.

Sample		TOC [%]	TN [%]	C/N
Macrophyte	WH	39.24	0.96	47.59
	MV	44.14	1.88	27.33
	HV	38.89	4.38	10.35
Algae	EA	36.61	4.46	9.57
	PA	49.33	6.83	8.43
	DA	42.30	7.30	6.76
Terrestrial standard sample	SRFA	52.34	0.67	91.14

Table S2. The optical parameters of UV-Vis absorption spectra of the macrophyte, algae and standard sample.

Sample		a_{254}/m^{-1}	a_{300}/m^{-1}	E3/E4	E2/E3	E4/E6	S_R
Macrophyte	WH	11.03	7.47	8.74	7.39	2.56	0.62
	MV	14.24	7.11	11.33	6.81	3.48	0.55
	HV	5.07	3.45	2.52	3.50	1.41	1.39
Algae	EA	3.82	0.45	1.01	2.70	2.00	2.19
	PA	5.99	4.15	2.13	3.15	1.77	1.56
	DA	6.65	1.10	2.17	2.60	1.89	1.60
Terrestrial standard sample	SRFA	52.22	29.26	4.39	4.31	6.36	0.76

Table S3. Proportions of three fluorescence components and characteristic values of fluorescence spectra from different DOM samples.

Sample		C1 (%)	C2 (%)	C3 (%)	FI	HIX	BIX
Macrophyte	WH	24.81	71.66	3.53	1.58	1.29	0.87
	MV	14.21	82.15	3.64	1.56	1.72	0.30
	HV	35.56	45.26	19.18	1.67	0.60	1.23
Algae	EA	4.11	81.01	14.88	2.52	0.38	0.70
	PA	8.87	77.24	13.89	2.19	0.26	0.81
	DA	10.21	74.95	14.84	2.27	0.34	0.86
Terrestrial standard sample	SRFA	51.89	3.94	44.17	1.10	14.51	0.44

Table S4. Intensity-weighted mean molecular parameters of different samples derived from assigned molecular formulae by Orbitrap MS.[a]

Sample	m/z_{wa}	n	C_{wa}	H_{wa}	O_{wa}	N_{wa}	S_{wa}	P_{wa}	H/C_{wa}	O/C_{wa}	DBE_{wa}	AI-mod_{wa}	$NOSC_{wa}$	I_{DEG}	CRAM (%)	MLBL (%)	CHO cpd. (%)	N cpd. (%)	S cpd. (%)	P cpd. (%)
**WH**	280.29	2819	12.91	16.34	6.64	0.10	0.03	0.01	1.30	0.51	5.80	0.28	-0.25	0.073	35.83	33.31	63.46	24.26	7.48	6.56
**MV**	287.83	2405	13.39	15.13	6.74	0.12	0.04	0.04	1.18	0.50	6.91	0.35	-0.15	0.714	39.38	32.18	59.33	24.07	10.94	6.99
**HV**	276.28	3706	13.54	21.55	5.17	0.33	0.04	0.12	1.56	0.40	3.98	0.15	-0.71	0.005	28.25	50.49	37.80	50.70	5.50	8.90
**EA**	295.42	2837	15.05	24.13	5.08	0.24	0.02	0.16	1.60	0.36	4.19	0.13	-0.87	0.003	24.71	58.90	35.50	40.61	9.45	21.29
**PA**	289.03	3340	14.66	23.37	5.05	0.17	0.02	0.18	1.58	0.36	4.16	0.14	-0.86	0.002	23.44	59.07	33.92	39.19	11.68	25.75
**DA**	250.87	4010	12.18	18.90	4.93	0.30	0.05	0.03	1.53	0.43	3.90	0.16	-0.58	0.017	28.48	47.66	33.52	48.33	17.41	8.20
**SRFA**	289.28	3857	13.70	15.17	6.74	0.06	0.01	0.02	1.12	0.50	7.15	0.37	-0.10	0.45	40.37	13.69	51.96	32.23	13.77	18.23

[a] Intensity weighted average values are displayed for mass-to-charge ratio (m/z_{wa}), number of carbon (C_{wa}), hydrogen (H_{wa}), oxygen (O_{wa}), nitrogen (N_{wa}), sulfur (S_{wa}), and phosphorus atoms (P_{wa}), hydrogen to carbon ratio (H/C_{wa}), oxygen to carbon ratio (O/C_{wa}), modified aromaticity index (AI-mod_{wa}), nominal oxidation state of carbon ($NOSC_{wa}$), double bond equivalent (DBE_{wa}), and degradation index of DOM (I_{DEG}). Abbreviations: MLBL, natural OM behaving as a labile substance value; CRAM, carboxyl-rich alicyclic molecules value; and n, number of identified molecules. The CHO cpd. (%) represent the percentage of compounds containing only C, H and O; the N, S, P cpd. (%) represent the percentage of nitrogenous compounds, sulfurated compounds, phosphorous compounds, respectively.

In previous studies (*Chem. Eng. J.* **2024**, 494, 152960), the molecular formula of SRFA has been identified as the majority of C, H and O atoms with a minimal presence of S atom. Additionally, the IHSS sample is distinguished by its minimal contribution of heteroatom formulae, including N, S and P atoms, within the subset of samples subjected to detailed analyses (mean molecular formula: $C_{17.3}H_{18.2}O_{7.4}N_{0.1}S_{0.05}$) (*Front. Earth Sci.* **2018**, 6, 138). Prior study has reported that the proportion of N-, S- and P-containing heteroatoms in SRFA can reach 25% in previous studies (*Environ. Sci. Technol.* **2018**, 52, 6771-6779). Similarly, the group

of commonly detected DOM formulae was previously represented by a majority of CHO, CHNO, and CHOS compounds. At present, improvements in resolution or analysis methods in most Orbitrap-MS studies may now uncover the characteristics of these heteroatoms (*Front. Earth Sci.* **2018**, 6, 138). The enhanced resolving power of modern Orbitrap instruments has enabled the detection of a diverse array of CHNO and CHOS compounds, along with modest quantities of CHOP and CHNOS formulae. For Suwannee River DOM 2R101N, the contribution rates of CHOS and CHOP formulas characterized by FT-ICR and Orbitrap MS to Bulk-DOM range from 9.4 to 12.8% (*Front. Earth Sci.* **2018**, 6, 138). Furthermore, decomposition methods and strategies have been proven to affect DOM MS data analysis, including in-house software, open-source platforms and commercial software (*Limnol. Oceanogr.:Methods* **2023**, 21, 320-333). These methods can yield different numbers of DOM molecules, and reveal the differences in O_{wa}, N_{wa}, and S_{wa}. Although there are differences in the alignment of MS results under disparate resolution strategies, especially regarding concentrated heteroatoms and low-intensity peaks, the overall composition of DOM molecules remains unchanged.

Table S5. Comparison of literature results on SRFA and DOM extraction from algae and macrophyte.[1]

Sample type	Sample name	Site	m/z_{wa}	n	C_{wa}	H_{wa}	O_{wa}	N_{wa}	S_{wa}	P_{wa}	H/C_{wa}	O/C_{wa}	DBE_{wa}	AI-mod$_{wa}$	$NOSC_{wa}$	I_{DEG}	CHO cpd. (%)	N cpd. (%)	S cpd. (%)	P cpd. (%)	Ref.
Algae	**Toxic cyanobacteria**	Lake Taihu	--	1269	--	--	--	--	--	--	1.56	0.28	--	--	--	--	20.09	26.87	53.03	--	*Water Res.* **2014**, 57, 280-294
	**[2]freshwater phytoplankton (Scenedesmus obliquus, Euglena mutabilis, and Euglena gracilis)**	Canadian Phycological Culture Center	--	--	--	--	--	--	--	--	1.30 - 1.54	0.23 - 0 .36	9.70 - 11.35	--	--	--	4.00 – 14.00*	41.00 – 93.00*	62.00 – 65.00*	--	*Anal. Bioanal. Chem.* **2016**, 408, 1891-1900
	**Cyanobacterial**	Lake Taihu	337.90	--	18.02	29.13	5.48	0.31	0.01	--	1.67	0.31	4.11	0.08	--	--	86.10	12.90	1.00	--	*Sci. Total Environ.* **2020**, 703, 134764
	**Algae**	Lake Chaohu	--	3141	--	--	--	--	--	--	--	--	6.69	0.16	--	--	34.67	51.57	19.32	--	*Environ. Sci. Technol.* **2021**, 55, 10811-10820
	**Cyanobacterial Microcystis**	Lake Taihu	335.00	3222	--	--	--	--	--	--	1.44	0.40	5.39	--	--	--	--	--	--	--	*Chemosphere* **2022**, 305, 135542
	**[2]Microcystis aeruginosa**	the Institute of Hydrobiology, Chinese Academy of Sciences	--	--	--	--	--	--	--	--	1.60	0.31	5.59	0.04	--	--	25.42	59.88	41.90	--	*Environ. Sci. Technol.* **2022**, 56, 13439-13448
	**Microcystis aeruginosa**	Lake Taihu	307.94	--	14.99	23.52	5.84	0.68	0.04	--	1.63	0.40	4.07	0.06	-0.68	--	59.60	36.60	4.30	--	*Chem. Eng. J.* **2024**, 494, 152960
	**Erhai algae**	Lake Erhai	358.82	3949	18.77	29.26	5.96	0.33	0.06	0.06	1.62	0.32	4.84	0.13	-0.87	0.07	44.87	40.59	9.57	9.01	This study

	**Puding algae**	Puding Karst Ecosystem Observation and Research Station, Chinese Academy of Sciences	375.67	5157	19.53	29.24	6.46	0.34	0.06	0.06	1.55	0.34	5.61	0.16	-0.77	0.06	45.32	41.17	11.19	9.04	This study
	**Dianchi algae**	Lake Dianchi	372.20	7817	18.48	28.39	6.92	0.33	0.19	0.01	1.59	0.39	4.96	0.11	-0.68	0.34	36.04	43.26	25.06	1.62	This study
**Macrophyte**	**Myriophyllum verticillatum L.**	Lake Taihu	380.60	--	17.18	21.05	9.28	0.24	0.04	--	1.29	0.55	7.27	0.20	--	--	79.90	15.70	3.70	--	*Sci. Total Environ.* **2020**, 703, 134764
	**Potamogeton Malaianus**	Lake Taihu	357.40	5843	--	--	--	--	--	--	1.15	0.44	8.12	--	--	--	--	--	--	--	*Chemosphere* **2022**, 305, 135542
	**Water hyacinth**	Puding Karst Ecosystem Observation and Research Station, Chinese Academy of Sciences	395.94	3177	17.35	18.65	10.45	0.03	0.03	0.01	1.14	0.61	8.54	0.31	0.15	0.40	78.06	12.78	6.70	2.90	This study
	**Myriophyllum verticillatum L.**		414.33	1514	18.89	17.95	10.44	0.02	0.03	0.04	1.02	0.56	10.44	0.40	0.16	0.84	77.74	9.51	8.98	5.02	This study
	**Hydrilla verticillata**		376.32	6831	19.45	30.29	6.57	0.36	0.05	0.01	1.61	0.35	4.99	0.12	-0.80	0.10	41.17	49.90	7.28	2.77	This study
**SRFA**	**[2]SRFA**	Suwannee River	--	--	--	--	--	--	--	--	1.13	0.36	11.93	--	--	--	--	1.51	10.44	--	*Anal. Bioanal. Chem.* **2016**, 408, 1891-1900
	**SRFA**	Suwannee River	416.54	--	19.18	18.97	10.39	0.04	0.02	--	1.05	0.55	10.21	0.36	0.05	--	94.90	3.34	1.74	--	*Chem. Eng. J.* **2024**, 494, 152960
	**SRFA**	Suwannee River	437.41	7759	20.52	20.02	10.57	0.05	0.02	0.01	1.03	0.52	11.04	0.41	0.07	0.68	73.00	20.08	6.64	4.55	This study

[1] The "--" sign means "not given". [2] Formulae of this article were identified by Orbitrap MS, and formulae of other articles were identified by FT-ICR-MS.

* The asterisks indicate that the data were obtained in positive ion mode, the unmarked default is negative ion mode.

Table S6. Distribution of O_3S and O_5S compounds in algae.

Instrument	Sample	O_3S		O_5S		O_3S and O_5S	
		Number	Proportion	Number	Proportion	Number	Proportion
**FT-ICR MS**	**EA**	12	0.30	51	1.29	**63**	1.60
	**PA**	13	0.25	98	1.90	**111**	2.15
	**DA**	33	0.42	181	2.32	**214**	2.74
**Orbitrap MS**	**EA**	26	0.92	48	1.69	**74**	2.61
	**PA**	26	0.78	76	2.28	**102**	3.05
	**DA**	72	1.80	102	2.54	**174**	4.34

Table S7. Composition of major subcategories of DOM in various samples analyzed by FT-ICR MS.

Sample	CHO	CHON	CHOS	CHOP	CHONP	Others		Total
						CHONS	CHOSP	
**WH**	2480 (78.06%)	404 (12.72%)	201 (6.33%)	78 (2.46%)	2 (0.06%)	0 (0%)	12 (0.38%)	3177
**MV**	1177 (77.74%)	138 (9.11%)	123 (8.12%)	57 (3.76%)	6 (0.40%)	0 (0%)	13 (0.86%)	1514
**HV**	2812 (41.17%)	3366 (49.28%)	464 (6.79%)	113 (1.65%)	43 (0.63%)	0 (0%)	33 (0.48%)	6831
**EA**	1772 (44.87%)	1553 (39.33%)	268 (6.79%)	**196 (4.96%)**	50 (1.27%)	0 (0%)	**110 (2.79%)**	3949
**PA**	2337 (45.32%)	1820 (35.29%)	296 (5.74%)	**358 (6.94%)**	65 (1.26%)	238 (4.62%)	43 (0.83%)	5157
**DA**	2817 (36.04%)	2939 (37.60%)	**1507 (19.28%)**	86 (1.10%)	16 (0.20%)	**427 (5.46%)**	25 (0.32%)	7817
**SRFA**	5664 (73.00%)	1329 (17.13%)	377 (4.86%)	58 (0.75%)	**193 (2.49%)**	36 (0.46%)	102 (1.31%)	7759

Table S8. Composition of major subcategories of DOM in various samples analyzed by Orbitrap MS.

Sample	CHO	CHON	CHOS	CHOP	CHONP	Others			Total
						CHONS	CHOSP	CHONSP	
**WH**	1789 (63.46%)	635 (22.53%)	197 (6.99%)	148 (5.25%)	36 (1.28%)	13 (0.46%)	1 (0.04%)	0 (0%)	2819
**MV**	1427 (59.33%)	553 (22.99%)	240 (9.98%)	154 (6.40%)	8 (0.33%)	17 (0.71%)	5 (0.21%)	1 (0.04%)	2405
**HV**	1401 (37.80%)	1776 (47.92%)	160 (4.32%)	261 (7.04 %)	64 (1.73%)	39 (1.05%)	5 (0.13 %)	0 (0%)	3706
**EA**	1007 (35.50%)	1019 (35.92%)	176 (6.20%)	442 (15.58%)	101 (3.56%)	31 (1.09%)	60 (2.11%)	1 (0.04%)	2837
**PA**	1133 (33.92%)	1053 (31.53%)	254 (7.60%)	548 (16.41%)	216 (6.47%)	40 (1.20%)	96 (2.87%)	0 (0%)	3340
**DA**	1344 (33.52%)	1646 (41.05%)	442 (11.02%)	286 (7.13%)	36 (0.90%)	249 (6.21%)	0 (0%)	7 (0.17%)	4010
**SRFA**	2004 (51.96%)	867 (22.48%)	201 (5.21%)	192 (4.98%)	263 (6.82%)	82 (2.13%)	217 (5.63%)	31 (0.80%)	3857

Table S9. Relative abundance (%) of van-Krevelen diagram classification from FT-ICR MS analysis of DOM from various sources.

Sample	Lipids	Proteins/Amino sugars	Carbohydrates	Unsaturated hydrocarbons	Lignins	Tannins	Condensed aromatic molecules
**WH**	128 (4.03%)	748 (23.57%)	67 (2.11%)	0 (0%)	1829 (57.62%)	313 (9.86%)	89 (2.80%)
**MV**	62 (4.13%)	213 (14.17%)	19 (1.26%)	0 (0%)	979 (65.14%)	164 (10.91%)	66 (4.39%)
**HV**	1462 (21.44%)	2051 (30.08%)	76 (1.11%)	2 (0.03%)	3030 (44.43%)	138 (2.02%)	60 (0.88%)
**EA**	849 (21.60%)	1447 (36.81%)	87 (2.21%)	17 (0.43%)	1498 (38.11%)	33 (0.84%)	0 (0%)
**PA**	1042 (20.26%)	1689 (32.85%)	135 (2.63%)	9 (0.18%)	2202 (42.82%)	64 (1.24%)	1 (0.02%)
**DA**	1186 (15.24%)	2737 (35.18%)	287 (3.69%)	3 (0.04%)	3168 (40.71%)	340 (4.37%)	60 (0.77%)
**SRFA**	90 (1.17%)	278 (3.61%)	28 (0.36%)	0 (0%)	5036 (65.42%)	998 (12.96%)	1268 (16.47%)

Table S10. Comparison of the classification derived from the van Krevelen diagram in the literature for DOM from algae, macrophyte and SRFA.[1]

Sample type	Sample name	Site	Lipids	Proteins/Amino sugars	Carbohydrates	Unsaturated hydrocarbons	Lignins	Tannins	Condensed aromatic molecules	References
**Algae**	**Cyanobacterial**	Lake Taihu	-- (56.80%)	-- (7.40%)	--	--	-- (15.00%)	--	--	*Sci. Total Environ.* **2020**, 703, 134764
	**Cyanobacterial Microcystis**	Lake Taihu	-- (8.60%)	-- (20.00%)	-- (15.00%)	--	-- (46.00%)	-- (10.00%)	--	*Chemosphere* **2022**, 305, 135542
	[2]**Microcystis aeruginosa**	the Institute of Hydrobiology, Chinese Academy of Sciences	-- (11.89%)	-- (21.73%)	-- (5.65%)	--	-- (46.20%)	-- (7.41%)	-- (7.12%)	*Environ. Sci. Technol.* **2022**, 56, 13439-13448
	**Algae**	Lake Dianchi	-- (18.00%)	-- (32.00%)	-- (3.50%)	-- (10.00%)	-- (34.00%)	-- (1.50%)	-- (1.00%)	*Environ. Pollut.* **2022**, 312, 119992
	**Microcystis aeruginosa**	Lake Taihu	230 (21.11%)	1126 (54.57%)	224 (1.38%)	0 (0.00 %)	1279 (22.23%)	121 (0.66%)	3 (0.01 %)	*Chem. Eng. J.* **2024**, 494, 152960
	**Erhai algae**	Lake Erhai	849 (21.60%)	1447 (36.81%)	87 (2.21%)	17 (0.43%)	1498 (38.11%)	33 (0.84%)	0 (0%)	This study
	**Puding algae**	Puding Karst Ecosystem Observation and Research Station, Chinese Academy of Sciences	1042 (20.26%)	1689 (32.85%)	135 (2.63%)	9 (0.18%)	2202 (42.82%)	64 (1.24%)	1 (0.02%)	This study
	**Dianchi algae**	Lake Dianchi	1186 (15.24%)	2737 (35.18%)	287 (3.69%)	3 (0.04%)	3168 (40.71%)	340 (4.37%)	60 (0.77%)	This study
**Macrophyte**	**Myriophyllum verticillatum**	Lake Taihu	-- (12.60%)	--	--	--	-- (50.60%)	-- (17.40%)	--	*Sci. Total Environ.* **2020**, 703, 134764

	**Potamogeton Malaianus**	Lake Taihu	-- (4.00%)	-- (8.00%)	-- (2.50%)	--	-- (68.00%)	-- (15.00%)	-- (1.50%)	*Chemosphere* **2022**, 305, 135542
	**Aquatic plant**	Lake Dianchi	-- (1.00%)	-- (5.00%)	-- (1.00%)	-- (1.00%)	-- (63.00%)	-- (20.00%)	-- (9.00%)	*Environ. Pollut.* **2022**, 312, 119992
	**Water hyacinth**	Puding Karst Ecosystem Observation and Research Station, Chinese Academy of Sciences	128 (4.03%)	748 (23.57%)	67 (2.11%)	0 (0%)	1829 (57.62%)	313 (9.86%)	89 (2.80%)	This study
	**Myriophyllum verticillatum**		62 (4.13%)	213 (14.17%)	19 (1.26%)	0 (0%)	979 (65.14%)	164 (10.91%)	66 (4.39%)	This study
	**Hydrilla verticillata**		1462 (21.44%)	2051 (30.08%)	76 (1.11%)	2 (0.03%)	3030 (44.43%)	138 (2.02%)	60 (0.88%)	This study
**SRFA**	[2]**SRFA**	Suwannee River	-- (0.59%)	-- (6.53%)	-- (0.76%)	-- (3.47%)	-- (77.19%)	-- (4.38%)	-- (5.83%)	*Anal. Bioanal. Chem.* **2016**, 408, 1891-1900
	**SRFA**	Suwannee River	47 (0.50%)	211 (0.95%)	27 (0.02%)	8 (0.00 %)	4741 (79.13%)	902 (13.51 %)	935 (5.90%)	*Chem. Eng. J.* **2024**, 494, 152960
	**SRFA**	Suwannee River	90 (1.17%)	278 (3.61%)	28 (0.36%)	0 (0.00%)	5036 (65.42%)	998 (12.96%)	1268 (16.47%)	This study

[1] The "--" sign means "not given".

[2] Formulae of this article were identified by Orbitrap MS, and formulae of other articles were identified by FT-ICR-MS.

Table S11. Relative abundance (%) of van-Krevelen diagram classification from Orbitrap MS analysis of DOM from various sources.

Sample	Lipids	Proteins/Amino sugars	Carbohydrates	Unsaturated hydrocarbons	Lignins	Tannins	Condensed aromatic molecules
**WH**	183 (6.50%)	660 (23.45%)	92 (3.27%)	0 (0%)	1447 (51.40%)	320 (11.37%)	113 (4.01%)
**MV**	144 (6.00%)	543 (22.63%)	84 (3.50%)	0 (0%)	1293 (53.88%)	243 (10.13%)	93 (3.88%)
**HV**	548 (14.84%)	1166 (31.57%)	145 (3.93%)	4 (0.11%)	1483 (40.16%)	225 (6.09%)	122 (3.30%)
**EA**	408 (14.54%)	1059 (37.73%)	174 (6.20%)	11 (0.39%)	1006 (35.84%)	114 (4.06%)	35 (1.25%)
**PA**	500 (15.10%)	1236 (37.33%)	211 (6.37%)	12 (0.36%)	1194 (36.06%)	130 (3.93%)	28 (0.85%)
**DA**	467 (11.72%)	1231 (30.89%)	190 (4.77%)	2 (0.05%)	1629 (40.88%)	354 (8.88%)	112 (2.81%)
**SRFA**	158 (4.12%)	229 (5.98%)	131 (3.42%)	3 (0.08%)	2242 (58.52%)	594 (15.51%)	474 (12.37%)

Table S12. Composition of major subcategories of LMW-DOM in various samples analyzed by Orbitrap MS.

Sample	CHO	CHON	CHOS	CHOP	CHONP	Others			Total	The proportion of compounds ≤ 200 Da to full compounds
						CHONS	CHOSP	CHONSP		
**WH**	155 (54.01%)	113 (39.37%)	13 (4.53%)	3 (1.05%)	0 (0%)	3 (1.05%)	0 (0%)	0 (0%)	287	10.18%
**MV**	165 (54.10%)	109 (35.74%)	22 (7.21%)	4 (1.31%)	0 (0%)	4 (1.31%)	1 (0.33%)	0 (0%)	305	12.68%
**HV**	154 (42.66%)	185 (51.25%)	14 (3.88%)	4 (1.11%)	0 (0%)	3 (0.83%)	1 (0.28%)	0 (0%)	361	9.74%
**EA**	159 (48.62%)	141 (43.12%)	13 (3.98%)	5 (1.53%)	0 (0%)	7 (2.14%)	2 (0.61%)	0 (0%)	327	11.53%
**PA**	158 (44.63%)	171 (48.31%)	14 (3.95%)	5 (1.41%)	1 (0.28%)	5 (1.41%)	0 (0%)	0 (0%)	354	10.60%
**DA**	165 (33.13%)	**210** (42.17%)	**66 (13.25%)**	**7** (1.41%)	1 (0.20%)	**49 (9.84%)**	0 (0%)	0 (0%)	**498**	12.42%
**SRFA**	181 (44.91%)	192 (47.64%)	18 (4.47%)	5 (1.24%)	2 (0.50%)	4 (0.99%)	1 (0.25%)	0 (0%)	403	10.45%

Table S13. Intensity-weighted mean molecular parameters of LMW-DOM in various samples analyzed by Orbitrap MS. [a]

Sample	m/z_{wa}	n	C_{wa}	H_{wa}	O_{wa}	N_{wa}	S_{wa}	P_{wa}	H/C_{wa}	O/C_{wa}	DBE_{wa}	AI-mod_{wa}	$NOSC_{wa}$	CRAM (%)	MLBL (%)	CHO cpd. (%)	N cpd. (%)	S cpd. (%)	P cpd. (%)
**WH**	157.87	287	7.57	9.62	3.53	0.06	0.001	0.001	1.28	0.49	3.79	0.35	-0.28	21.25	34.84	54.01	40.42	5.57	1.05
**MV**	155.34	305	7.51	9.50	3.39	0.09	0.008	0.0002	1.29	0.47	3.80	0.36	-0.32	20.33	36.72	54.10	37.05	8.85	1.64
**HV**	162.50	361	8.00	11.01	3.19	0.30	0.006	0.001	1.39	0.41	3.64	0.30	-0.44	21.33	35.73	42.66	52.08	4.99	1.39
**EA**	158.42	327	7.81	11.09	3.18	0.15	0.01	0.003	1.42	0.43	3.34	0.27	-0.50	23.24	40.67	48.62	45.26	6.73	2.14
**PA**	162.45	354	8.12	11.51	3.13	0.22	0.006	0.001	1.42	0.41	3.48	0.28	-0.51	23.16	41.53	44.63	50.00	5.37	1.69
**DA**	161.34	498	7.78	10.85	3.30	0.25	0.02	0.001	1.41	0.44	3.48	0.27	-0.42	21.49	37.95	33.13	52.21	23.09	1.61
**SRFA**	155.81	403	7.59	8.89	3.39	0.11	0.002	0.0003	1.19	0.47	4.19	0.40	-0.20	21.34	34.74	44.91	49.13	5.71	1.99

[a] Intensity weighted average values are displayed for mass-to-charge ratio (m/z_{wa}), number of carbon (C_{wa}), hydrogen (H_{wa}), oxygen (O_{wa}), nitrogen (N_{wa}), sulfur (S_{wa}), and phosphorus atoms (P_{wa}), hydrogen to carbon ratio (H/C_{wa}), oxygen to carbon ratio (O/C_{wa}), modified aromaticity index (AI-mod_{wa}), nominal oxidation state of carbon ($NOSC_{wa}$), double bond equivalent (DBE_{wa}), and degradation index of DOM (I_{DEG}). Abbreviations: MLBL, natural OM behaving as a labile substance value; CRAM, carboxyl-rich alicyclic molecules value; and n, number of identified molecules. The CHO cpd. (%) represent the percentage of compounds containing only C, H and O; the N, S, P cpd. (%) represent the percentage of nitrogenous compounds, sulfurated compounds, phosphorous compounds, respectively.

Table S14. Distribution of S-containing LMW-DOM in DA.

Species	CHOS					CHONS			
	CHOS	O_2S	O_3S	O_4S	O_5S	CHONS	NO_2S	NO_3S	NO_4S
Number	66	17	25	18	6	49	20	18	11

Table S15. Distribution of P-containing LMW-DOM in macrophyte, algae, and SRFA *via* Orbitrap MS, where figures represent numbers.

Sample	P-containing LMW-DOM										
WH	CHOP				CHONP				CHOSP		
	Total	O_3P	O_4P	O_5P	Total	NO_2P	NO_3P	NO_4P	Total	SO_2P	SO_4P
	3	1	1	1	0	0	0	0	0	0	0
MV	CHOP				CHONP				CHOSP		
	Total	O_3P	O_4P	O_5P	Total	NO_2P	NO_3P	NO_4P	Total	SO_2P	SO_4P
	4	0	2	2	0	0	0	0	1	0	1
HV	CHOP				CHONP				CHOSP		
	Total	O_3P	O_4P	O_5P	Total	NO_2P	NO_3P	NO_4P	Total	SO_2P	SO_4P
	4	0	4	0	0	0	0	0	1	0	1
EA	CHOP				CHONP				CHOSP		
	Total	O_3P	O_5P	O_4P	Total	NO_2P	NO_3P	NO_4P	Total	SO_2P	SO_4P
	5	0	1	4	0	0	0	0	2	0	2
PA	CHOP				CHONP				CHOSP		
	Total	O_3P	O_4P	O_5P	Total	NO_2P	NO_3P	NO_4P	Total	SO_2P	SO_4P
	5	1	2	2	1	0	0	1	0	0	0
DA	CHOP				CHONP				CHOSP		
	Total	O_3P	O_4P	O_5P	Total	NO_2P	NO_3P	NO_4P	Total	SO_2P	SO_4P
	7	0	3	4	1	0	0	1	0	0	0
SRFA	CHOP				CHONP				CHOSP		
	Total	O_3P	O_4P	O_5P	Total	NO_2P	NO_3P	NO_4P	Total	SO_2P	SO_4P
	5	1	2	2	2	1	1	0	1	1	0

Table S16. Relative abundance (%) of van-Krevelen diagram-derived classification from Orbitrap MS analysis of LMW-DOM.

Sample	Lipids	Proteins/Amino sugars	Carbohydrates	Unsaturated hydrocarbons	Lignins	Tannins	Condensed aromatic molecules
**WH**	30 (10.49%)	48 (16.78%)	21 (7.34%)	0 (0%)	135 (47.20%)	31 (10.84%)	21 (7.34%)
**MV**	29 (9.60%)	52 (17.22%)	28 (9.27%)	0 (0%)	133 (44.04%)	36 (11.92%)	24 (7.95%)
**HV**	40 (11.20%)	64 (17.93%)	21 (5.88%)	0 (0%)	171 (47.90%)	34 (9.52%)	27 (7.56%)
**EA**	37 (11.38%)	66 (20.31%)	28 (8.62%)	0 (0%)	145 (44.62%)	31 (9.54%)	18 (5.54%)
**PA**	43 (12.39%)	69 (19.88%)	28 (8.07%)	0 (0%)	153 (44.09%)	37 (10.66%)	17 (4.90%)
**DA**	47 (9.61%)	**95** (19.43%)	38 (7.77%)	0 (0%)	**222** (45.40%)	52 (10.63%)	35 (7.16%)
**SRFA**	41 (10.33%)	66 (16.62%)	28 (7.05%)	0 (0%)	166 (41.81%)	53 (13.35%)	**43 (10.83%)**

For each sample, the molecular composition of LMW-DOM was dominated by lignin and protein (**Table S16**). Previous studies have reported that regardless of the molecular weight and source of DOM, lignin is the most important component, accounting for approximately 50–80% of total compound (*Chemosphere* **2022**, 305, 135542). Additionally, the percentage of each compound showed a high degree of similarity in different samples (**Figure S6**; **Table S16**). The unsaturated hydrocarbons were not detected due to their weak polarity. The characterization of LMW-DOM was clearly different from the molecular composition profile of Bulk-DOM (**Table S16**). The relative content of each compound was as follows: lignins (41.5–48.0%) > proteins (16.5–20.5%) > tannins (9.5–13.5%) > lipids (9.5–12.5%) > condensed aromatic molecules (4.5–11.0%) > carbohydrates (5.5–9.5%). This means that the specificity of terrestrial and endogenous compounds in Bulk-DOM from macrophytes and algae gradually disappeared, while the molecular structures of LMW-DOM converged.

Table S17. The possible structures and names of DOM with high strength.

Entry	m/z	formula	O/C	H/C	Name	Structure	Class	Source
1	285.20706	$C_{16}H_{30}O_4$	0.25	1.88	Thapsic acid	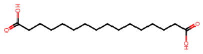	lipid-like	EA
2	289.07177	$C_{15}H_{14}O_6$	0.40	0.93	D-(+)-Catechin	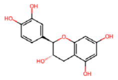	lignin-like	MV
3	295.04593	$C_{13}H_{12}O_8$	0.62	0.92	Caffeoylmalic acid	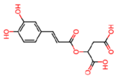	lignin-like	WH
4	301.03537	$C_{15}H_{10}O_7$	0.47	0.67	Quercetin	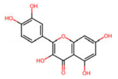	condensed aromatic molecules	MV
5	301.0565	$C_{12}H_{14}O_9$	0.75	1.17	Pyrogallol-2-O-glucuronide	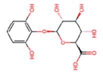	tannin-like	MV
6	303.21763	$C_{16}H_{32}O_5$	0.31	2.00	Aleuritic Acid	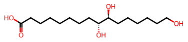	protein-like	EA
7	307.19141	$C_{18}H_{28}O_4$	0.22	1.56	Albocyclin	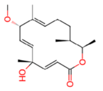	lipid-like	EA
8	309.06157	$C_{14}H_{14}O_8$	0.57	1.00	Methyl 3,4,5-triacetoxybenzoate	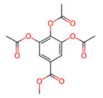	lignin-like	WH
9	309.20709	$C_{18}H_{30}O_4$	0.22	1.67	decanediol dimethacrylate	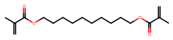	lipid-like	HV/DA/PA
10	311.07721	$C_{14}H_{16}O_8$	0.57	1.14	4-{[(2E)-3-(3,4-Dihydroxyphenyl)-2-propenoyl]oxy}-2,3-dihydroxy-2-methylbutanoic acid	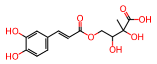	lignin-like	WH
11	313.05651	$C_{13}H_{14}O_9$	0.69	1.08	1-Salicylate glucuronide	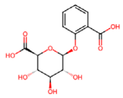	tannin-like	WH
12	315.07214	$C_{13}H_{16}O_9$	0.69	1.23	5-(β-D-Glucopyranosyloxy)-2-hydroxybenzoic acid	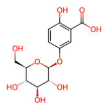	tannin-like	MV
13	317.23327	$C_{17}H_{34}O_5$	0.29	2.00	Pentaerythritol laurate	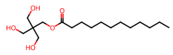	lipid-like	EA
14	323.1864	$C_{18}H_{28}O_5$	0.28	1.56	Methyl 7-[5-oxo-3-(tetrahydro-2H-pyran-2-yloxy)-1-cyclopenten-1-yl]heptanoate	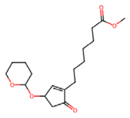	lipid-like	DA
15	325.09282	$C_{15}H_{18}O_8$	0.53	1.20	Melilotoside	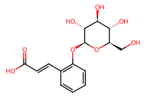	lignin-like	SRFA
16	325.18417	$C_{18}H_{30}O_3S_1$	0.17	1.67	4-Dodecylbenzenesulfonic acid	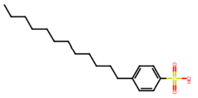	lipid-like	SRFA
17	325.20195	$C_{18}H_{30}O_5$	0.28	1.67	Oxydi-2,1-ethanediyl dicyclohexanecarboxylate	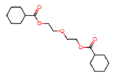	lipid-like	HV/EA/DA/PA

18	327.2176	$C_{18}H_{32}O_5$	0.28	1.78	Diethyl 5,5′-tetrahydrofuran-2,5-diyldipentanoate	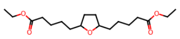	lipid-like	HV/EA/DA/PA
19	329.23325	$C_{18}H_{34}O_5$	0.28	1.89	2-Ethylhexyl 3-methoxypropyl adipate	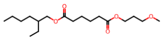	lipid-like	HV/DA
20	331.06704	$C_{13}H_{16}O_{10}$	0.77	1.23	Glucogallin	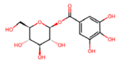	tannin-like	MV
21	333.20713	$C_{20}H_{30}O_4$	0.20	1.50	Dihexyl phthalate	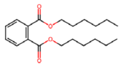	lipid-like	EA
22	337.0565	$C_{15}H_{14}O_9$	0.60	0.93	(2R,3R)-2,3-Dihydroxy-1-(2,4,6-trihydroxyphenyl)-3-(3,4,5-trihydroxyphenyl)-1-propanone	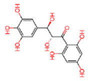	lignin-like	WH
23	339.1085	$C_{16}H_{20}O_8$	0.50	1.25	trans-isoeugenol-O-glucuronide	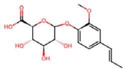	lignin-like	SRFA
24	343.21252	$C_{18}H_{32}O_6$	0.33	1.78	Trivalerin	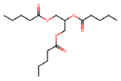	protein-like	HV/DA/PA
25	351.21761	$C_{20}H_{32}O_5$	0.25	1.60	(−)-Prostaglandin E2	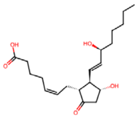	lipid-like	EA
26	353.05138	$C_{15}H_{14}O_{10}$	0.67	0.93	3-Carboxy-2,3-dideoxy-4-O-[(2Z)-3-(3,4-dihydroxyphenyl)-2-propenoyl]pentaric acid	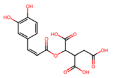	lignin-like	WH
27	353.0878	$C_{16}H_{18}O_9$	0.56	1.13	Chlorogenic acid	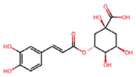	lignin-like	MV
28	355.06703	$C_{15}H_{16}O_{10}$	0.67	1.07	5-[(E)-2-Carboxyvinyl]-2-hydroxyphenyl β-D-glucopyranosiduronic acid	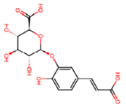	lignin-like	WH
29	367.06703	$C_{16}H_{16}O_{10}$	0.63	1.00	2,3-Dideoxy-4-O-[(2E)-3-(3,4-dihydroxyphenyl)-2-propenoyl]-3-(methoxycarbonyl)pentaric acid	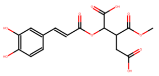	lignin-like	WH
30	367.10326	$C_{17}H_{20}O_9$	0.53	1.18	3-Feruloylquinic acid	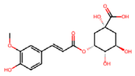	lignin-like	SRFA
31	371.0619	$C_{15}H_{16}O_{11}$	0.73	1.07	2-O-caffeoylglucaric acid	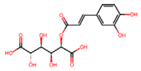	tannin-like	WH
32	381.1189	$C_{18}H_{22}O_9$	0.50	1.22	Methyl (1R,3R,4S,5R)-1,3,4-trihydroxy-5-{[(2E)-3-(4-hydroxy-3-methoxyphenyl)-2-propenoyl]oxy}cyclohexanecarboxylate	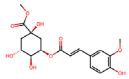	lignin-like	SRFA
33	385.0776	$C_{16}H_{18}O_{11}$	0.69	1.13	2-(E)-O-feruloyl-D-galactaric acid	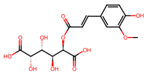	tannin-like	WH
34	395.1346	$C_{19}H_{24}O_9$	0.47	1.26	1,5-Anhydro-1-(2-sec-butyl-5,7-dihydroxy-4-oxo-4H-chromen-8-yl)hexitol	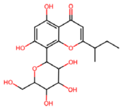	lignin-like	SRFA
35	441.0827	$C_{22}H_{18}O_{10}$	0.45	0.82	(−)-Epicatechin-3-O-gallate	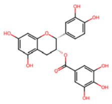	lignin-like	MV

36	447.09317	$C_{21}H_{20}O_{11}$	0.52	0.95	Astragalin	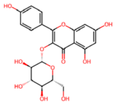	lignin-like	MV
37	483.0779	$C_{20}H_{20}O_{14}$	0.70	1.00	1,6-bis-O-galloyl-β-D-glucose	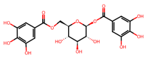	tannin-like	MV
38	569.0936	$C_{27}H_{22}O_{14}$	0.52	0.81	5-galloylquercetin-3-O-α-L-arabinofuranoside	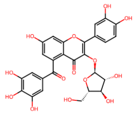	lignin-like	MV
39	579.13541	$C_{26}H_{28}O_{15}$	0.58	1.08	Leucoside	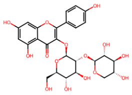	lignin-like	MV
40	609.14597	$C_{27}H_{30}O_{16}$	0.59	1.11	Rutin	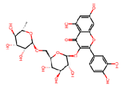	lignin-like	MV
41	635.08883	$C_{27}H_{24}O_{18}$	0.67	0.89	1,3,6-Trigalloyl glucose	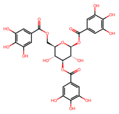	lignin-like	MV
42	695.26523	$C_{34}H_{49}O_{11}S_1P_1$	0.32	1.44	({1-[(Cyclohexyloxy)sulfonyl]-4-(3-phenoxyphenyl)butyl}phosphoryl)bis(oxymethylene) bis(2,2-dimethylpropanoate)	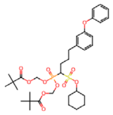	lignin-like	EA

Table S18. The possible structures and names of LMW-DOM with high strength.

Entry	M/Z	formula	O/C	H/C	Name	Structure	Source
1	101.06024	$C_5H_{10}O_2$	0.40	2.00	Valeric acid	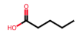	MV/DA/SRFA
2	109.02894	$C_6H_6O_2$	0.33	1.00	Hydroquinone	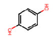	MV/SRFA
3	111.00821	$C_5H_4O_3$	0.60	0.80	2-Furoic acid	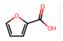	SRFA
4	113.02386	$C_5H_6O_3$	0.60	1.20	Glutaric anhydride	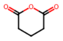	SRFA
5	113.06024	$C_6H_{10}O_2$	0.33	1.67	Acetonylacetone	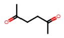	WH
6	115.00312	$C_4H_4O_4$	1.00	1.00	Fumaric acid	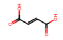	WH/MV
7	115.03951	$C_5H_8O_3$	0.60	1.60	Levulinic acid	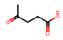	EA/DA/SRFA
8	115.07589	$C_6H_{12}O_2$	0.33	2.00	1-Hexanoic acid	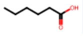	MV/EA/DA/SRFA
9	121.02894	$C_7H_6O_2$	0.29	0.86	Benzoic acid	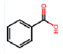	WH/MV/HV/EA/PA/SRFA
10	125.02386	$C_6H_6O_3$	0.50	1.00	Pyrogallol	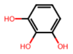	MV/SRFA
11	127.03951	$C_6H_8O_3$	0.50	1.33	Furaneol	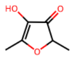	SRFA
12	128.03475	$C_5H_7N_1O_3$	0.60	1.40	L-Pyroglutamic acid	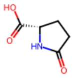	EA
13	129.01877	$C_5H_6O_4$	0.80	1.20	Itaconic acid	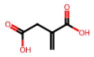	WH
14	129.05516	$C_6H_{10}O_3$	0.50	1.67	Ketoleucine	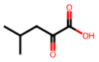	HV/EA/DA/SRFA
15	129.09154	$C_7H_{14}O_2$	0.29	2.00	n-Heptanoic acid	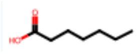	MV/EA/DA/SRFA
16	130.08679	$C_6H_{13}N_1O_2$	0.33	2.17	Aminocaproic acid	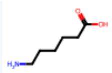	MV/HV
17	131.03442	$C_5H_8O_4$	0.80	1.60	Glutaric acid	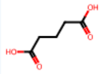	DA
18	131.07081	$C_6H_{12}O_3$	0.50	2.00	Leucic acid	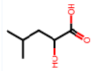	MV/HV/DA
19	135.04459	$C_8H_8O_2$	0.25	1.00	4-Methoxybenzaldehyde	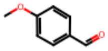	WH/MV/DA
20	137.02386	$C_7H_6O_3$	0.43	0.86	Salicylic acid	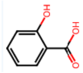	WH/MV/HV/DA/SRFA
21	139.03951	$C_7H_8O_3$	0.43	1.14	3-Methoxycatechol	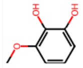	SRFA
22	143.07081	$C_7H_{12}O_3$	0.43	1.71	Ethyl 2-methylacetoacetate	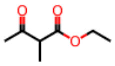	DA
23	145.05007	$C_6H_{10}O_4$	0.67	1.67	Adipic acid	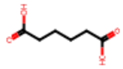	EA
24	145.08646	$C_7H_{14}O_3$	0.43	2.00	n-Butyl lactate	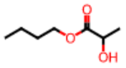	WH/HV

25	147.02934	$C_5H_8O_5$	1.00	1.60	Ribonolactone	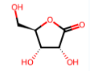	WH
26	147.04459	$C_9H_8O_2$	0.22	0.89	Cinnamic acid	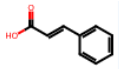	MV
27	147.06572	$C_6H_{12}O_4$	0.67	2.00	Methyl 3,3-dimethoxypropionate	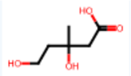	MV
28	149.02386	$C_8H_6O_3$	0.38	0.75	Phenylglyoxylic acid	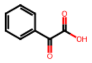	WH
29	149.06024	$C_9H_{10}O_2$	0.22	1.11	3-Phenylpropanoic acid	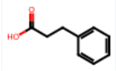	WH
30	151.00312	$C_7H_4O_4$	0.57	0.57	5-Hydroxy-1,3-benzodioxol-2-one	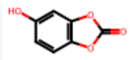	MV
31	151.03951	$C_8H_8O_3$	0.38	1.00	Vanillin	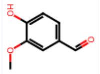	WH/MV/HV/DA/SRFA
32	153.01877	$C_7H_6O_4$	0.57	0.86	2,3-Dihydroxybenzoic acid	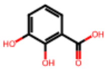	WH/MV/HV/SRFA
33	153.09154	$C_9H_{14}O_2$	0.22	1.56	Methyl 2-octynoate	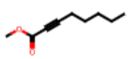	EA/PA
34	155.10719	$C_9H_{16}O_2$	0.22	1.78	Apricolin	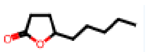	WH
35	157.05007	$C_7H_{10}O_4$	0.57	1.43	Dimethyl itaconate	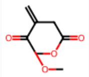	HV/DA
36	157.08646	$C_8H_{14}O_3$	0.38	1.75	Ethyl 2-ethylacetoacetate	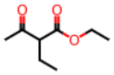	EA/DA
37	158.08170	$C_7H_{13}N_1O_3$	0.43	1.86	N-Acetylvaline	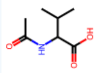	HV/DA/PA
38	159.06572	$C_7H_{12}O_4$	0.57	1.71	Pimelic acid	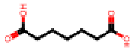	HV/EA/DA/PA
39	161.02386	$C_9H_6O_3$	0.33	0.67	Umbelliferone	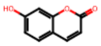	WH
40	161.04499	$C_6H_{10}O_5$	0.83	1.67	Levoglucosan	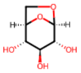	DA
41	161.08137	$C_7H_{14}O_4$	0.57	2.00	2,3-O-isopropylidene-L-threitol	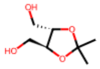	WH/MV
42	163.03951	$C_9H_8O_3$	0.33	0.89	(E)-p-coumaric acid	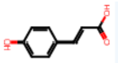	WH/HV/EA/DA/SRFA
43	164.07114	$C_9H_{11}N_1O_2$	0.22	1.22	DL-Phenylalanine	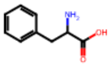	MV
44	165.01877	$C_8H_6O_4$	0.50	0.75	Phthalic acid	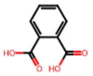	WH/MV/HV/EA/DA/PA/SRFA
45	165.05516	$C_9H_{10}O_3$	0.33	1.11	Apocynin	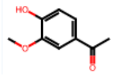	WH/DA/SRFA
46	167.03442	$C_8H_8O_4$	0.50	1.00	Vanillic acid	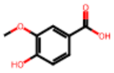	WH/MV/SRFA

47	169.08646	$C_9H_{14}O_3$	0.33	1.56	Ethyl 2-oxocyclohexanecarboxylate	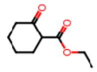	HV/EA/DA/PA
48	169.12284	$C_{1}0H_{18}O_2$	0.20	1.80	γ-Decanolactone	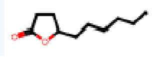	HV/PA
49	171.06572	$C_8H_{12}O_4$	0.50	1.50	Diethyl fumarate	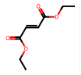	WH/MV/HV/DA/PA
50	171.10211	$C_9H_{16}O_3$	0.33	1.78	Butyl levulinate	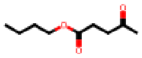	WH/HV/EA/DA/PA
51	172.09735	$C_8H_{15}N_1O_3$	0.38	1.88	N-Acetyl-L-leucine	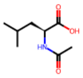	WH/MV/HV/DA
52	173.00860	$C_6H_6O_6$	1.00	1.00	(Z)-Aconitic Acid	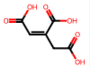	WH
53	173.04499	$C_7H_{10}O_5$	0.71	1.43	(−)-Shikimic acid	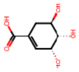	DA/SRFA
54	173.08137	$C_8H_{14}O_4$	0.50	1.75	Suberic acid	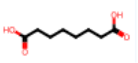	WH/MV/HV/EA/DA/PA
55	175.06064	$C_7H_{12}O_5$	0.71	1.71	Diacetin	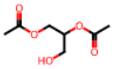	WH/EA/PA
56	175.09702	$C_8H_{16}O_4$	0.50	2.00	Ethyl diglycol acetate	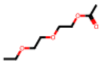	WH/HV/DA
57	177.01877	$C_9H_6O_4$	0.44	0.67	6,7-Dihydroxycoumarin	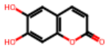	WH/MV/SRFA
58	179.03442	$C_9H_8O_4$	0.44	0.89	trans-caffeic acid	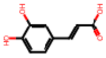	MV/HV/DA/SRFA
59	181.01369	$C_8H_6O_5$	0.63	0.75	3,5-Dicarboxyphenol	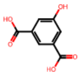	SRFA
60	181.05007	$C_9H_{10}O_4$	0.44	1.11	Homovanillic acid	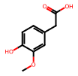	WH/SRFA
61	181.08646	$C_{10}H_{14}O_3$	0.30	1.40	Mephenesin	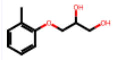	EA/DA/PA
62	183.06572	$C_9H_{12}O_4$	0.44	1.33	Antiarol	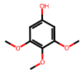	EA/DA
63	183.10211	$C_{10}H_{16}O_3$	0.30	1.60	Ethyl 2-Cyclohexanoneacetate	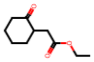	DA/PA
64	185.08137	$C_9H_{14}O_4$	0.44	1.56	Diethyl ethylidenemalonate	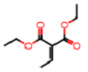	HV/EA/DA
65	185.11776	$C_{10}H_{18}O_3$	0.30	1.80	Queen Bee Acid	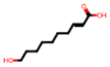	DA
66	186.11300	$C_9H_{17}N_1O_3$	0.33	1.89	(R)-3-(2-Amino-2-oxoethyl)-5-methylhexanoic acid	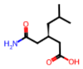	HV
67	187.06064	$C_8H_{12}O_5$	0.63	1.50	2,3-Isopropylidene-D-ribonolactone	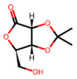	EA/DA
68	187.09702	$C_9H_{16}O_4$	0.44	1.78	Azelaic acid	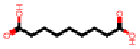	WH/MV

69	188.09227	$C_8H_{15}N_1O_4$	0.50	1.88	N-Butoxycarbonyl-L-alanine	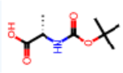	HV
70	189.07629	$C_8H_{14}O_5$	0.63	1.75	Ethyl malate	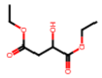	WH/MV/DA
71	191.03442	$C_{10}H_8O_4$	0.40	0.80	Scopoletin	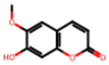	SRFA
72	191.05555	$C_7H_{12}O_6$	0.86	1.71	Sedoheptulosan	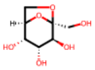	MV
73	192.06605	$C_{10}H_{11}N_1O_3$	0.30	1.10	Phenaceturic Acid	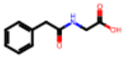	HV
74	193.01369	$C_9H_6O_5$	0.56	0.67	4,5,7-Trihydroxycoumarin	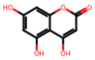	SRFA
75	193.05007	$C_{10}H_{10}O_4$	0.40	1.00	(E)-Ferulic acid	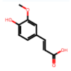	MV
76	195.02934	$C_9H_8O_5$	0.56	0.89	4-Methoxyphthalic acid	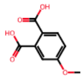	WH/SRFA
77	195.10211	$C_{11}H_{16}O_3$	0.27	1.45	3,4-Dimethoxybenzenepropanol	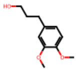	EA/PA
78	197.00860	$C_8H_6O_6$	0.75	0.75	2,5-Dihydroxyterephthalic acid	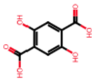	SRFA
79	197.04499	$C_9H_{10}O_5$	0.56	1.11	Syringic acid	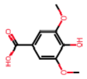	MV/SRFA
80	197.08137	$C_{10}H_{14}O_4$	0.40	1.40	Guaifenesin	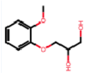	EA/DA/PA
81	197.11776	$C_{11}H_{18}O_3$	0.27	1.64	Ethyl 3-cyclohexyl-3-oxopropanoate	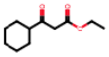	HV/PA
